# Correction: Naturally Occurring Deletions of Hunchback Binding Sites in the *Even-Skipped* Stripe 3+7 Enhancer

**DOI:** 10.1371/journal.pone.0099548

**Published:** 2014-06-04

**Authors:** The PLOS ONE Staff

## Notice of Republication

This article was republished on May 13, 2014, to correct the ordering of Figures 3 and 4, formatting in the “Testing the Effects of a Segregating Deletion on Adult Phenotypes” section, and the orientation of Tables 2, 3, and 4 in the PDF. Please download this article again to view the correct version. The originally published, uncorrected article and the republished, corrected article are provided here for reference.

## Supporting Information

File S1 Originally published, uncorrected article.
(PDF)

File S2 Republished, corrected article.
(PDF)
