# Supplementary figures and images for: Naturally Occurring Deletions of Hunchback Binding Sites in the Even-Skipped Stripe 3+7 Enhancer
Source: PLoS One. 2014 May 1;9(5):e91924. doi: 10.1371/journal.pone.0091924 (PMC4006794; doi:10.1371/journal.pone.0091924)

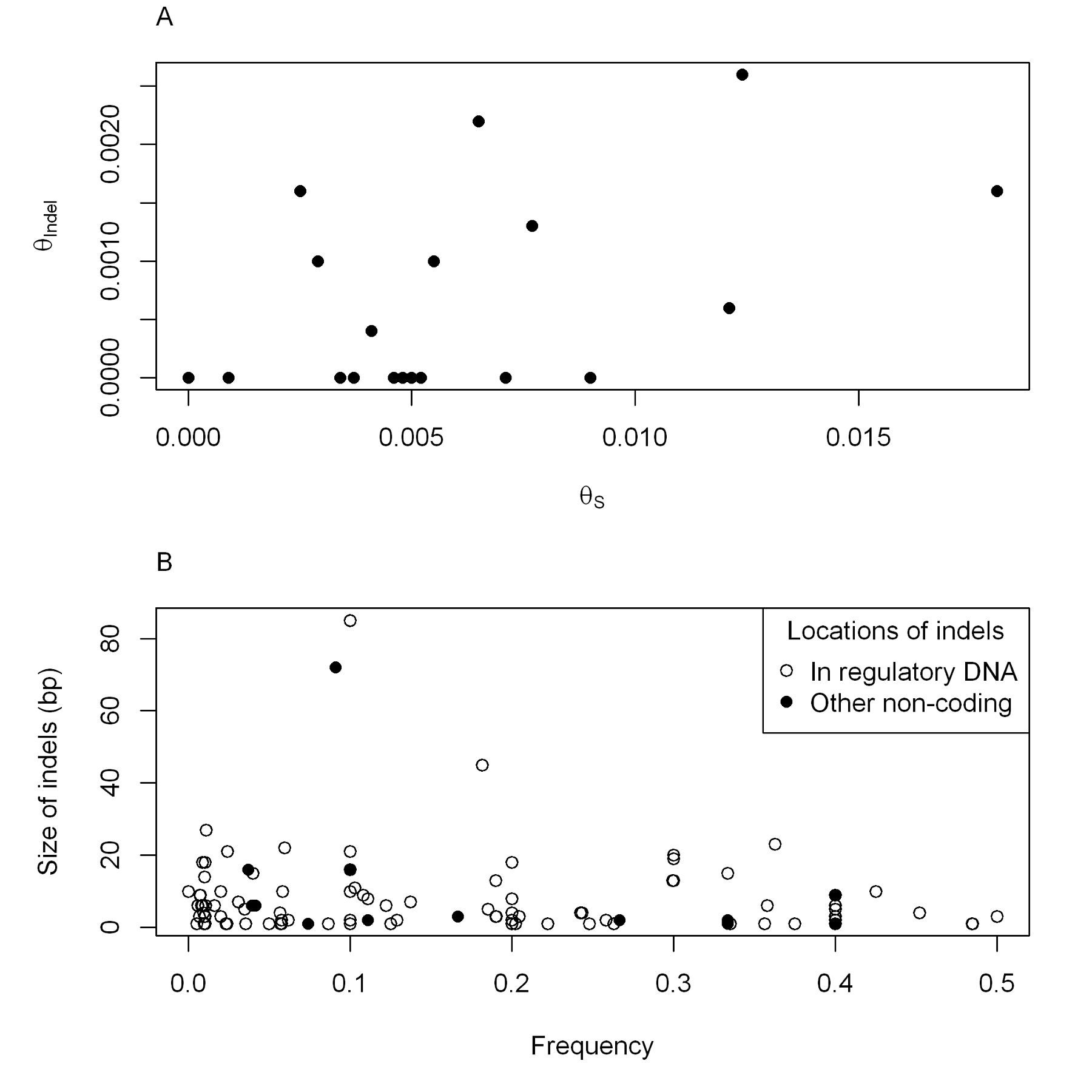

Supplement: Figure S1 — Constraints on SNPs and indels in regulatory DNA. A) The relationship between single base and indel polymorphism (summarized with θ) in 19 enhancers and promoters in D. melanogaster. Many of the characterized enhancers have no indels, and sit therefore at Y = 0. B) Size and frequency of indels in characterized regulatory DNA and proximate promoters (dark circles) vs. indels in non-coding regions (open circles) around two developmental genes (hairy and EGFR). (TIFF) [file pone.0091924.s001.tif]

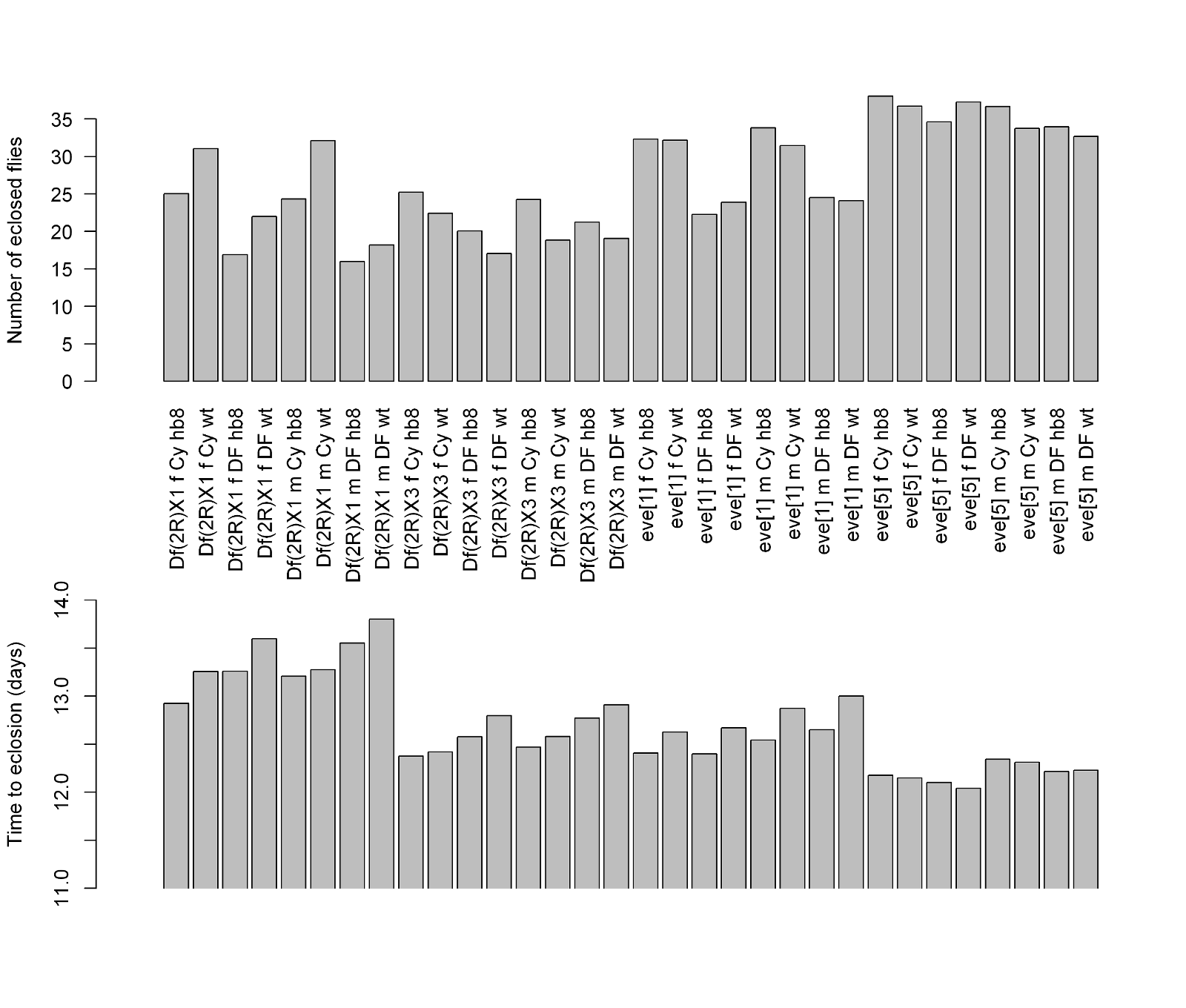

Supplement: Figure S3 — Effects of Hb8Δ alleles on viability (above) and developmental time (below). Represented are least square mean estimates for combination of eve mutation (alleles and deficiency chromosomes), balancer (Cy) or mutation carrying chromosome (DF) and sex. Developmental time was estimated as the time to eclosion. (TIFF) [file pone.0091924.s003.tif]
