# Supplementary material for: Naturally Occurring Deletions of Hunchback Binding Sites in the Even-Skipped Stripe 3+7 Enhancer
Source: PLoS One. 2014 May 1;9(5):e91924. doi: 10.1371/journal.pone.0091924 (PMC4006794; doi:10.1371/journal.pone.0091924)
Supplement: Table S3 — Conservation of binding sites in the eve stripe 3+7 enhancer. Transcription factor binding site numbering of sites follows Stanjovic et al 1989, Small et al 1996 and Yan et al 1996. Hb binding site 16 is on the opposite strand. Full species names and accession numbers are listed in material and methods. (*) indicate bases shared by two overlapping binding sites. (N/A) sites not identified in these species. Full species names and accession numbers are listed in material and methods. (*) indicate bases shared by two overlapping binding sites. (N/A) sites not identified in these species. The order reflects approximately phylogenetic relationship available on http://insects. eugenes.org/species. There is length variation in T stretch between Kni5 and Hb11c; extra 1 and 2 bases in D. sim and D. gri respectively. As these are monomorphic stretches the core binding sites are presumably not affected. (DOC) [file pone.0091924.s006.doc]

Table S3. (part 1) Conservation of binding sites in the *eve* stripe 3+7 enhancer.

|  | Hb16^1^ | Hb15 | Kni1 | Hb14a | Hb14b |
| --- | --- | --- | --- | --- | --- |
| *D.mel* | CCACTAAAAAT | CTTTTTATTGG | AGAAAACTAGATCA | TTTTTTGTTT | ATTTTTGTGC |
| *D.sim* | ........... | ........... | .............. | .......... | .......... |
| *D.sech* | ........... | ........... | .............. | .......... | .......... |
| *D.yak* | ........... | ........... | .............. | .......... | .......... |
| *D.ere* | ........... | ........... | .............. | .........T | .......... |
| *D.ana* | ........... | ........... | .............. | .......... | .......... |
| *D.pse* | ........... | ........... | .............. | .......... | .......... |
| *D.per* | ........... | ........... | .............. | .......... | .......... |
| *D.vir* | ........... | ........... | N/A | N/A | N/A |
| *D.gri* | ........... | ........... | N/A | N/A | N/A |
| *D.moj* | ........... | ........... | N/A | N/A | N/A |
|  |  |  |  |  | ---------* |

|  | Kni2 | Hb14c | Kni3 |
| --- | --- | --- | --- |
| *D.mel* | CCCG---GTGCTCT-------CTTT | CT-------CTTTACGG | TGGCCGCGTT-------------------CCCAT |
| *D.sim* | ....---.......-------.... | ..-------........ | ..........-------------------..... |
| *D.sech* | ....---.......-------.... | ..-------........ | ..........-------------------..... |
| *D.yak* | ....---.......-------.... | ..-------........ | .....TT...TCC---ATTTCGTCATTTTT.... |
| *D.ere* | ....--TT......-------.... | ..-------........ | ..........TCCATTATTTCGTCATTTTT...C |
| *D.ana* | .T.TGCTT----.G-------.... | .G-------.....T.. | ...T..----------------TCGCTTTT.... |
| *D.pse* | ...TGCCC......CCTTATG.... | ..CCTTATG.....TT. | ...T..-A..TCCA-------------TTT.... |
| *D.per* | ...TGCTC......CCTTATG.... | ..CCTTATG.....TT. | ...T..-A..TCCA-------TTTCCATTTT... |
| *D.vir* | N/A | N/A | N/A |
| *D.gri* | N/A | N/A | N/A |
| *D.moj* | N/A | N/A | N/A |
|  | *----------************* | *************---- |  |

Transcription factor binding site numbering of sites follows Stanjovic et al 1989, Small et al 1996 and Yan et al 1996. Hb binding site 16 is on the opposite strand. Full species names and accession numbers are listed in material and methods. (*) indicate bases shared by two overlapping binding sites. (N/A) sites not identified in these species.

Table S3. (part 2)

|  | Hb13 | Hb12 | Stat1 | Kni4 | Hb11a | Hb11b |
| --- | --- | --- | --- | --- | --- | --- |
| *D.mel* | TTC--TTTGTTC | TTTTTATGG | GTTCGCGGAA---C | AGGAAAGTAGATC | TTTTTTGTTC | TTTTTTCG-C |
| *D.sim* | ...--....... | ......... | ..........---. | ............. | .......... | ........-. |
| *D.sech* | ...--....... | ......... | ..........---. | ............. | .........T | ........-. |
| *D.yak* | ...--.....G. | ......... | ..........---. | .A........... | .......... | ........-. |
| *D.ere* | ...--.....G. | ......... | ..........---. | .A........... | .......... | ........-. |
| *D.ana* | ...--A.....T | ........T | ..........---. | ............. | .........T | ........-. |
| *D.pse* | C..--A.....T | ......... | ..........---T | ............. | .......... | ........-. |
| *D.per* | C..--A.....T | ......... | ..........---T | ............. | .......... | ........-. |
| *D.vir* | ..T--G.....G | ......... | T.........TTC. | .....T....... | .........T | ........G. |
| *D.gri* | .T.--A.....T | ......... | C.........ACCG | .....T....... | .........T | ........-. |
| *D.moj* | G..TT......A | ......... | ..........TTTT | .....T....... | .........T | ........-. |
|  |  |  |  |  |  | ---------* |

|  | Kni5^2^ | Hb11c^2^ | Stat2 | Hb10 | Hb9 | Hb8 |
| --- | --- | --- | --- | --- | --- | --- |
| *D.mel* | CTGCGCTAGTT | TTTTTTTCCC | TTTCCCCGAA-C | TTTTTTAATTC | TTTTTAAGA | GTTTTTACGA |
| *D.sim* | ........... | .......... | ..........-. | ........... | ......... | .......... |
| *D.sech* | ........T.. | .........G | .....G....-. | ........... | ......... | .......... |
| *D.yak* | ......C.C.. | ..C....... | ..........-. | ........... | ......... | A......T.. |
| *D.ere* | ......C.... | ..C.G..... | G.........-. | ........... | ......... | A......T.. |
| *D.ana* | ........... | .......... | ......A...-. | ........... | N/A | N/A |
| *D.pse* | ........... | .......... | ..........-. | ........... | ......... | A......T.. |
| *D.per* | ........... | .......... | ..........-. | ........... | ......... | A......T.. |
| *D.vir* | ........... | .......... | ..........G. | ........... | .......-- | A......TT. |
| *D.gri* | ....C...... | .......... | ..........G. | ........... | .......C. | A......TT. |
| *D.moj* | ........... | .......... | ..........G. | ........... | ......CC. | A......TT. |
|  | *--------** | **---***** | *****------- |  |  |  |

Full species names and accession numbers are listed in material and methods. (*) indicate bases shared by two overlapping binding sites. (N/A) sites not identified in these species. The order reflects approximately phylogenetic relationship available on http://insects.eugenes.org/species. There is length variation in T stretch between Kni5 and Hb11c; extra 1 and 2 bases in *D. sim* and *D. gri* respectively. As these are monomorphic stretches the core binding sites are presumably not affected.
