# Supplementary material for: Naturally Occurring Deletions of Hunchback Binding Sites in the Even-Skipped Stripe 3+7 Enhancer
Source: PLoS One. 2014 May 1;9(5):e91924. doi: 10.1371/journal.pone.0091924 (PMC4006794; doi:10.1371/journal.pone.0091924)
Supplement: Table S4 — Little evolutionary conservation of a putative sloppy-paired site in the eve stripe 3+7 enhancer. Full species names and accession numbers are listed in material and methods. Orthology of the sloppy-paired binding site region was determined by colinearity of binding sites in the stripe 3+7 region, were Hb8 and Hb9 flank the sloppy-paired binding site. Fewer than 50 bp separated Hb8 and Hb9 in all species. The exception is D. ananassae, were Hb8 was not detected. (DOC) [file pone.0091924.s007.doc]

Table S4. Little evolutionary conservation of a putative sloppy-paired site in the *eve* stripe 3+7 enhancer.

| Species | Predicted slp1 site |
| --- | --- |
| *D.mel* | GTTTGTTTGTGTTTGTTTGT----------------CC |
| *D.sim* | GTTTGT----GTTTGTTTGT----------------CC |
| *D.sech* | GTTTGT----GTTTGTTTGT----------------CC |
| *D.yak* | G--CTTGTGTTTCTGCTTGT----------------CC |
| *D.ere* | G----TTTGTGTTCGTCTCT----------------GG |
| *D.ana* | N/A |
| *D.pse* | GTTCTTTTCTCT--GTGTTTTCTGTTCTGTTCTTTTCC |
| *D.per* | GTTCTTTTCTCTCTGTGTTTTCTGTTCTGTTCTTTTCC |
| *D.vir* | N/A |
| *D.gri* | N/A |
| *D.moj* | N/A |

Full species names and accession numbers are listed in material and methods. Orthology of the sloppy-paired binding site region was determined by colinearity of binding sites in the stripe 3+7 region, were Hb8 and Hb9 flank the sloppy-paired binding site. Fewer than 50 bp separated Hb8 and Hb9 in all species. The exception is *D. ananassae*, were Hb8 was not detected.
