# Supplementary material for: Naturally Occurring Deletions of Hunchback Binding Sites in the Even-Skipped Stripe 3+7 Enhancer
Source: PLoS One. 2014 May 1;9(5):e91924. doi: 10.1371/journal.pone.0091924 (PMC4006794; doi:10.1371/journal.pone.0091924)
Supplement: Table S5 — The presence of the Hb8Δ in a world wide sample of populations. A deletion specific primer, annealing to regions joined by the mutation was used in a PCR on pooled DNA (100 individuals) from each of the 51 populations. Pop: Population. (DOC) [file pone.0091924.s008.doc]

Table S5. The presence of the *Hb8Δ* in a world wide sample of populations.

| Pop | Country | *Hb8Δ* | Pop | Country | *Hb8Δ* | Pop | Country | *Hb8Δ* |
| --- | --- | --- | --- | --- | --- | --- | --- | --- |
| F01 | Mexico | + | F19 | Madagascar | + | F36 | French Guiana | - |
| F02 | Mexico | + | F20 | Madagascar | + | F37 | Guadeloupe | - |
| F03 | Greece | + | F21 | Mayotte Is. | + | F38 | Virgin Island | + |
| F04 | Mexico | + | F22 | Mauritius | + | F39 | Uruguay | + |
| F05 | Mexico | + | F23 | Swaziland | + | F40 | Morocco | + |
| F07 | France | + | F24 | Congo | - | F41 | Congo | - |
| F08 | France | + | F25 | Congo | + | F42 | Tanzania | + |
| F09 | Egypt | + | F26 | Seychelles | + | F43 | Ivory Coast | + |
| F10 | Egypt | + | F27 | Martinique | + | F44 | South Africa | + |
| F11 | Iraq | + | F28 | Georgia (USA) | + | F45 | China | + |
| F12 | Sao Tome | + | F29 | Brazil | + | F46 | India | + |
| F13 | Niger | + | F30 | French Polynesia | + | F47 | India | + |
| F14 | Kenya | + | F31 | India | - | F48 | Australia | + |
| F15 | Benin | + | F32 | Massachusetts | + | F49 | Canada | + |
| F16 | Benin | + | F33 | Massachusetts | + | F52 | Israel | - |
| F17 | Ivory Coast | + | F34 | Massachusetts | + | F53 | Japan | - |
| F18 | Ivory Coast | + | F35 | USA | + | F54 | Japan | - |

A deletion specific primer, annealing to regions joined by the mutation was used in a PCR on pooled DNA (100 individuals) from each of the 51 populations.

Pop: Population.
