# Supplementary material for: Naturally Occurring Deletions of Hunchback Binding Sites in the Even-Skipped Stripe 3+7 Enhancer
Source: PLoS One. 2014 May 1;9(5):e91924. doi: 10.1371/journal.pone.0091924 (PMC4006794; doi:10.1371/journal.pone.0091924)
Supplement: Table S6 — Mixed model ANOVA on eve stripe positioning. (DOC) [file pone.0091924.s009.doc]

Table S6. Mixed model ANOVA on *eve* stripe positioning.

|  |  | | Anterior boundary | | | | Posterior boundary | | | |
| --- | --- | --- | --- | --- | --- | --- | --- | --- | --- | --- |
| *eve*  stripe | Term/  Var.Comp*^a^* | | d.f. | *F*-value | *P* | d.f. | | *F*-value | *P* |  |
| 1 | DV | | 4,15 | 7.38 | 1.7E-03 | 4,15 | | 6.66 | 2.7E-03 |  |
|  | Time | | 1,922 | 35.59 | 3.5E-09 | 1,922 | | 4.51 | 3.4E-02 |  |
|  | T X DV | | 4,922 | 3.08 | 1.6E-02 | 4,922 | | 3.08 | 1.6E-02 |  |
|  | *V_Line(DV)_* |  | | 0.084 | 7.4E-03 |  | | 0.104 | 6.2E-03 |  |
|  | *V_error_* | |  | 0.392 | 1.5E-102 |  | | 0.373 | 1.5E-102 |  |
| 2 | DV | | 4,15 | 6.25 | 3.6E-03 | 4,15 | | 4.21 | 1.8E-02 |  |
|  | Time | | 1,922 | 0.87 | 3.5E-01 | 1,922 | | 65.03 | 2.3E-15 |  |
|  | T X DV | | 4,922 | 1.79 | 1.3E-01 | 4,922 | | 2.23 | 6.4E-02 |  |
|  | *V_Line(DV)_* |  | | 0.145 | 5.1E-03 |  | | 0.131 | 5.4E-03 |  |
|  | *V_error_* | |  | 0.354 | 1.5E-102 |  | | 0.355 | 1.5E-102 |  |
| 3 | DV | | 4,15 | 2.48 | 8.8E-02 | 4,15 | | 1.27 | 3.3E-01 |  |
|  | Time | | 1,922 | 53.31 | 6.1E-13 | 1,922 | | 132.6 | 9.2E-29 |  |
|  | T X DV | | 4,922 | 1.4 | 2.3E-01 | 4,922 | | 0.95 | 4.3E-01 |  |
|  | *V_Line(DV)_* |  | | 0.136 | 4.9E-03 |  | | 0.154 | 4.6E-03 |  |
|  | *V_error_* | |  | 0.301 | 1.5E-102 |  | | 0.296 | 1.5E-102 |  |
| 4 | DV | | 4,15 | 0.26 | 9.0E-01 | 4,15 | | 0.32 | 8.6E-01 |  |
|  | Time | | 1,922 | 126.83 | 1.2E-27 | 1,922 | | 232.29 | 6.0E-47 |  |
|  | T X DV | | 4,922 | 0.49 | 7.4E-01 | 4,922 | | 0.49 | 7.4E-01 |  |
|  | *V_Line(DV)_* |  | | 0.205 | 4.1E-03 |  | | 0.215 | 4.1E-03 |  |
|  | *V_error_* | |  | 0.274 | 1.5E-102 |  | | 0.293 | 1.5E-102 |  |
| 5 | DV | | 4,15 | 0.93 | 4.7E-01 | 4,15 | | 2.9 | 5.8E-02 |  |
|  | Time | | 1,922 | 205.87 | 2.7E-42 | 1,922 | | 270.61 | 1.6E-53 |  |
|  | T X DV | | 4,922 | 0.76 | 5.5E-01 | 4,922 | | 1.32 | 2.6E-01 |  |
|  | *V_Line(DV)_* |  | | 0.168 | 4.5E-03 |  | | 0.174 | 4.6E-03 |  |
|  | *V_error_* | |  | 0.323 | 1.5E-102 |  | | 0.362 | 1.4E-102 |  |
| 6 | DV | | 4,15 | 2.96 | 5.5E-02 | 4,15 | | 5.84 | 4.9E-03 |  |
|  | Time | | 1,922 | 146.62 | 2.0E-31 | 1,922 | | 227.93 | 3.5E-46 |  |
|  | T X DV | | 4,922 | 2.52 | 4.0E-02 | 4,922 | | 3.83 | 4.3E-03 |  |
|  | *V_Line(DV)_* |  | | 0.145 | 5.1E-03 |  | | 0.107 | 6.5E-03 |  |
|  | *V_error_* | |  | 0.399 | 1.4E-102 |  | | 0.477 | 1.4E-102 |  |
| 7 | DV | | 4,15 | 4.01 | 2.1E-02 | 4,15 | | 3.85 | 2.4E-02 |  |
|  | Time | | 1,922 | 216.93 | 3.0E-44 | 1,922 | | 293.82 | 2.2E-57 |  |
|  | T X DV | | 4,922 | 5.08 | 4.8E-04 | 4,922 | | 5.39 | 2.7E-04 |  |
|  | *V_Line(DV)_* |  | | 0.137 | 6.4E-03 |  | | 0.13 | 7.4E-03 |  |
|  | *V_error_* | |  | 0.589 | 1.4E-102 |  | | 0.697 | 1.4E-102 |  |
| 1 to 7*^b^* | DV | | 4,15 | 8.06 | 1.1E-03 |  | |  |  |  |
|  | Time | | 1,922 | 270.11 | 2.0E-53 |  | |  |  |  |
|  | T X DV | | 4,922 | 4.9 | 6.5E-04 |  | |  |  |  |
|  | *V_Line(DV)_* |  | | 0.282 | 6.7E-03 |  | |  |  |  |
|  | *V_error_* | |  | 1.207 | 1.5E-102 |  | |  |  |  |

a. The explanatory variables, either Time or Dorsal Ventral index, and variance components (multiplied by 1000) for the random terms. Significance of the VC’s was estimated by the *z*-function.

b. Relative distance between *eve* stripes 1 and 7.
