# Supplementary material for: Correction: Naturally Occurring Deletions of Hunchback Binding Sites in the Even-Skipped Stripe 3+7 Enhancer
Source: PLoS One. 2014 Jun 4;9(6):e99548. doi: 10.1371/journal.pone.0099548 (PMC4045991; doi:10.1371/journal.pone.0099548)
Supplement: File S1 — Originally published, uncorrected article. (PDF) [file pone.0099548.s001.pdf]

OPEN ACCESS Freely available online

PLOS ONE

Naturally Occurring Deletions of Hunchback Binding Sites in the *Even-Skipped* Stripe 3+7 Enhancer

Arnar Palsson[1,2,3,4]*, Natalia Wesolowska[4,5], Sigrún Reynisdóttir[1,2], Michael Z. Ludwig[4], Martin Kreitman[4]

1 Faculty of Life and Environmental Sciences, University of Iceland, Reykjavik, Iceland, 2 Institute of Biology, University of Iceland, Reykjavik, Iceland, 3 Biomedical Center, University of Iceland, Reykjavik, Iceland, 4 Department of Ecology and Evolution, University of Chicago, Chicago, Illinois, United States of America, 5 Cell Biology and Biophysics Unit, European Molecular Biology Laboratory (EMBL), Heidelberg, Germany

Abstract

Changes in regulatory DNA contribute to phenotypic differences within and between taxa. Comparative studies show that many transcription factor binding sites (TFBS) are conserved between species whereas functional studies reveal that some mutations segregating within species alter TFBS function. Consistently, in this analysis of 13 regulatory elements in *Drosophila melanogaster* populations, single base and insertion/deletion polymorphism are rare in characterized regulatory elements. Experimentally defined TFBS are nearly devoid of segregating mutations and, as has been shown before, are quite conserved. For instance 8 of 11 Hunchback binding sites in the stripe 3+7 enhancer of *even-skipped* are conserved between *D. melanogaster* and *Drosophila virilis*. Oddly, we found a 72 bp deletion that removes one of these binding sites (Hb8), segregating within *D. melanogaster*. Furthermore, a 45 bp deletion polymorphism in the spacer between the stripe 3+7 and stripe 2 enhancers, removes another predicted Hunchback site. These two deletions are separated by ~250 bp, sit on distinct haplotypes, and segregate at appreciable frequency. The *Hb8Δ* is at 5 to 35% frequency in the new world, but also shows cosmopolitan distribution. There is depletion of sequence variation on the *Hb8Δ*-carrying haplotype. Quantitative genetic tests indicate that *Hb8Δ* affects developmental time, but not viability of offspring. The Eve expression pattern differs between inbred lines, but the stripe 3 and 7 boundaries seem unaffected by *Hb8Δ*. The data reveal segregating variation in regulatory elements, which may reflect evolutionary turnover of characterized TFBS due to drift or co-evolution.

Citation: Palsson A, Wesolowska N, Reynisdóttir S, Ludwig MZ, Kreitman M (2014) Naturally Occurring Deletions of Hunchback Binding Sites in the *Even-Skipped* Stripe 3+7 Enhancer. PLoS ONE 9(5): e91924. doi:10.1371/journal.pone.0091924

Editor: Alan M. Moses, University of Toronto, Canada

Received September 19, 2013; **Accepted** February 18, 2014; **Published** May 1, 2014

Copyright: © 2014 Palsson et al. This is an open-access article distributed under the terms of the Creative Commons Attribution License, which permits unrestricted use, distribution, and reproduction in any medium, provided the original author and source are credited.

Funding: This project was funded by the National Institutes of Health (Grant R01 GM61001 to M.K. and M.Z.L.), Icelandic research fund (IRF 070260021 to A.P.), Marie Curie international reintegration grant (MIRG-CT-2007-046510 to A.P.) and the University of Iceland research fund to A.P. The funders had no role in study design, data collection and analysis, decision to publish, or preparation of the manuscript.

Competing Interests: Arnar Palsson is an PLOS ONE editor. This does not alter the authors' adherence to all the PLOS ONE policies on sharing data and materials.

* E-mail: apalsson@hi.is

Introduction

Evolution of Transcriptional Regulatory Sequences

The molecular basis for phenotypic divergence and standing variation is often attributed to differences in the regulation of transcription[1–3]. The mechanistic principles of regulatory DNA and factor structure and function such as; multiple transcription factor binding sites (TFBS), TFBS motif degeneracy, cooperativity and number of trans factors [3,4] and interactions between transcription factors (TFs), enhancers and promoters [5,6] impose unique rules on their evolution. Regulatory DNA has no single "active-site", since most regions consist of multiple transcription factor binding sites. Evolutionary analyses of experimentally verified TFBS demonstrate examples of conservation, but also reveal evolutionary turnover of TFBS, were some sites are lost and others gained [7–9].

It has been postulated that selection mainly acts on the transcriptional output of a gene (timing, location and amount) and does not preserve individual TFBS [10,11]. That is, changes in TFBS and even losses are permitted, if the transcriptional output is preserved. Such models of stabilizing selection acting on transcriptional output can account for both loss of functional binding sites and evolutionary fine-tuning of regulatory elements [12]. They also suggest that positive selection may sometimes play a role, acting on compensatory mutations in *cis* or *trans*. Several studies [13–16] have investigated the evolutionary origin of TFBS, including co-evolution within regulatory sequences. From first principles one would predict both co-evolution in *cis* (promoters, regulatory modules, more distantly located signals like insulators) and co-evolution of sequence elements with the *trans* environment (abundance of transcription factor, mediator or holenzyme components). The model of *trans* co-evolution is corroborated by studies of between-species hybrids [17], which *e.g.* reveal mis-expression of genes in hybrids of *D. melanogaster* and *D. simulans*, two closely related species, most likely due to species-specific *cis-trans* compensatory evolution. Also, genome-wide changes in *cis* elements of co-expressed genes in two distantly related yeast species document the co-evolution of the TF repertoire of an organism and the regulatory elements of coordinately expressed genes [18,19]. Numerical models show how mutation and drift can generate binding sites, and predictably that selection can speed up fixation of new TFBS [20]. Crucially, functional polymorphism (both single nucleotide polymorphism: SNPs or insertion/deletion polymorphism: indels) in human enhancers, are shaped by positive selection [21].

Insertion and Deletion Polymorphism in Regulatory DNA

Population genetics studies have largely neglected indels, perhaps because they represent a minority of segregating variation in most genomes [22]. Deletion polymorphism in the intergenic region of *Adh* in *Drosophila pseudoobscura* does not conform to neutral evolution, but exhibits signatures of purifying selection, *i.e.* deletion (but not insertion) polymorphism was removed from introns over time [23]. On a larger scale, Comeron and Kreitman [24] revealed a bias in the insertion and deletion frequency distribution in *D. melanogaster* populations. While deletion events were more common and on average longer, insertions were at significantly higher frequency. This may reflect both mutational bias (because the mechanisms causing deletions are different from those causing insertions) and a difference in selection pressures, with purifying selection keeping a large fraction of deletions at low frequency in the population [24,25]. Ometto *et al.* [25], on the other hand, also concluded that weak positive selection might increase the population frequency of some insertions, which is supported by a genome-wide study in *D. melanogaster* [26]. Population genetic analyses of Bicoid response genes in *D. melanogaster* revealed single nucleotide polymorphism (SNPs) in 13 of 85 predicted Bicoid binding sites [27]. Most notable was the high frequency of SNP and indel polymorphism in the *Orthodenticle* (*Otd*) early head enhancer. These polymorphisms clustered on two haplotypes, both at intermediate frequency. Transgene tests showed that the *Otd* haplotypes differ in transcriptional output [27]. Similarly, studies of the *Endo16* promoter and other sea-urchin enhancers [28–30] show that many TFBS are affected by segregating indel variation. In particular, in *Endo16* two rare insertions affect the same part of the promoter. One of these generated a functional repressor module [29].

Enhancers of Eve as a Model of Regulatory Evolution

Early embryonic development in *D. melanogaster* is regulated by numerous genes through a complex network of activation and repression, resulting in segmental boundaries along the embryo length [31–35]. The accurate temporal and spatial expression of these genes is mainly achieved by integration of multiple TFs and their binding to regulatory sequences. Some regulatory functions (required for a given expression pattern) are aggregated in distinct modules like the *eve* stripe 2 enhancer (*s2e*) and the stripe 3 and 7 enhancer (*s3+7e*). These experimentally verified "minimal" enhancers [36,37] suffice to generate 4–7 cell-wide Eve stripes in early development. Not all regulatory sequences contain modular enhancers, and often spacer sequences (separating regulatory modules) have function, meaning that the length of these sequences matters for proper function of flanking *cis*-modules [10,38].

The cumulative effect of nucleotide changes in *s2e* between species is a turnover of functional motifs within enhancers [8,11,39]. Notably, the *s2e* from *D. erecta* is less effective than *s2e* from the more distantly related *D. pseudoobscura* at complementing a deletion of the *s2e* in *D. melanogaster* [11]. Quantitative analysis of the amount of Eve in stripe 2 illustrated the functional deficiency of the *D. erecta s2e* in the *D. melanogaster* genetic background. This means that, for a given enhancer, the spatial and temporal features of the expression pattern are highly conserved, but the quantity of gene product probably less so. The expression level of developmentally-specific gene products may exhibit changes over evolutionary time, possibly reflecting "developmental system drift" [40,41].

The aim of the current study was to gauge the level of polymorphism in the well-characterized regulatory regions in *D. melanogaster,* with particular focus on insertion and deletion polymorphism. Consistent with other studies and evolutionary theory, SNP and indel-polymorphism are rare in TFBS. However we find two peculiarly large and common deletions in and close to the *eve* stripe 3 and 7 enhancer. Both deletions remove binding sites for Hunchback, prompting analysis of the genetics and phylogeography of one of those polymorphisms and its potential phenotypic effects. The data provide insights into the nature of variation segregating in *cis*-regulatory elements.

Materials and Methods

Flies and Populations

Several populations of flies where studied. The population genetic surveys were done on collections of inbred lines derived from North Carolina, collected in 2000 and 2005 [27,42], and a Costa Rican sample from Peter Andolfatto, made isogenic for the second chromosome by three generations of crosses. Walter Eanes provided DNA from thirteen US East coast populations [43]; a total of 380 individuals used to test for clinal variation in the *eve* region. Jean-Claude Walser provided a sample of 46 cosmopolitan populations [44], in which DNA from 100 lines in each population was pooled.

PCR, DNA Sequencing and Genotyping

Primers were designed with primer 3 version 0.3 (frodo.wi.mit.edu [45]) for 13 well-characterized early developmental enhancers or promoters and several other non-coding regions (see Table S1). The regions studied were several parts of the *eve* locus (the *late element, s2e, s3+7e,* and the promoter, along with two spacer sequences), *Kruppel* promoter and *CD1, salm* wing blade enhancer, *ems* abdominal enhancer, *en* regulatory region and promoters *ofAntp, Ubx-bxd, tll, Act57B, RpL29/CG30390* and *RpL30.* The sequence variation in those regions was assessed by PCR followed directly by DNA sequencing. PCR was done as before [46] with Takara Taq and MJ Tetrad machines on 96 well plates. Products where purified by Qiagen purification columns or Exo-sap. DNA sequencing was done on purified PCR products, with the forward and reverse primer using Applied biosystems reagents. The ethanol purified reaction products where run in the University of Chicago sequencing facility or the ABI sequencing machine at the Institute of Biology, University of Iceland.

The deletion of the Hb8 site in *s3+7e* (see below) and the wild type allele were genotyped with PCR using allele-specific primers (Table S1). We ran separate reactions for both alleles on individuals from the East coast sample and on bulk DNA samples from the cosmopolitan sample. This was used to infer geographic distribution of specific variants, but does of course not yield information about frequency. All sequences were submitted as Popset data to NCBI (accession numbers: KJ465109–KJ465866), except two alignments that were shorter than 200 bp (provided in fasta format as Supporting information S1 and S2).

Population Genetic Analysis

Metrics of population genetics (S, π, θ, Haplotype number) were calculated for SNPs and indels with Tassel vs. 2.1 (www.maizegenetics.net [47]), either for individual regions or as a sliding window for the haplotype analysis. Tassel was also used to calculate LD, and R (www.r-project.org version 12.3 [48]) for testing of contingency tables. DNAsp vs. 4.1 (www.ub.edu/dnasp [49]) was also used to test for deviations of Tajima's D and Fu and Li's estimators. Furthermore Hudson's haplotype test [50] (utilizing the ms program and the psub option) was used to test for positive selection in four *eve* regions.

Phylogenetic Shadowing

A 2 kb region surrounding the stripe 3+7 minimum enhancer was blasted against the 12 finished genomes (insects.eugenes.org/species), and the orthologous regions extracted (except *D. willistoni* which did not return a significant blast hit). The Drosophila species (abbreviated) and contig names and locations are listed; *D. melanogaster* (*D. mel*), release 4, *D. simulans* (*D.sim*) chromosome 2R, bases 4491595 to 4494659, *D. sechellia* (*D. sech*) scaffold 359, bases 7623 to 10695, *D. erecta* (*D. ere*) scaffold 4929, bases 8504394 to 8507885, *D. yakuba* (*D. yak*) chromosome 2L, bases 18628840 to 18632292, *D. ananassae* (*D. ana*) scaffold 13266, bases 15371395 to 15373454, *D. pseudoobscura* (*D. pse*) chromosome 3, bases 10879010 to 10881069, *D. persimilis* (*D. per*) scaffold 4, bases 6230662 to 6232721, *D. virilis* (*D. vir*) scaffold 12875, bases 1335449 to 1337479, *D. grimshawi* (*D. gri*) scaffold 15245, bases 9663295 to 9665324, *D. mojavensis* (*D. moj*) scaffold 6496, bases 4426987 to 4430428. The sequences were aligned with MAVID (baboon.math.berkeley.edu/mavid [51]). Divergence in these sequences is considerable, requiring manual curating in Genedoc (www.psc.edu/biomed/genedoc [52]), with special devotion to characterized TFBS from redfly.ccr.buffalo.edu [53] and ORegAnno [54]. In addition two additional Hb sites (Hb15 and Hb16) found by Stanojovic *et al.* [55] and two Stat binding sites discovered by Yan *et al.* [32] were included. We found that the *D. melanogaster* Stat binding sites differ from the genomic sequence, probably due to sequencing error (Stat-1 was reported to start with an **A** and stat-2 was reported as **G**TTCCCCGAA**A**, highlighted bases differ).

We also used (jaspar.genereg.net [56]) to predict Hb binding sites (score above 6) in the ~8000 bp upstream of *eve,* in *D. melanogaster, D. sechellia, D. yakuba* and *D. pseudoobscura.* Based on multiple alignments from Mavid, and Multiz alignments from the Santa Cruz genome browser (downloaded in December 2013), we mapped predicted Hb binding sites in orthologous and more rapidly evolving regions.

Testing the Effects of a Segregating Deletion on Adult Phenotypes

A set of 20–60 healthy inbred lines from NC [46] were used for the two experiments conducted to test the effects of a 72 bp deletion within *s3+7e* (called *Hb8Δ,* see below) on viability and developmental time. The first was a set of controlled crosses to lines deficient for *eve*, and the second was phenotyping of 60 genotyped inbred lines. All fly-rearing took place on cornmeal food at constant temperature, 25°C.

We first crossed the inbred lines to four stocks with characterized *eve* mutations. Ten inbred lines, homozygous for each allele (*Hb8Δ* or *wt*) were crossed to each *eve* mutant. The Bloomington stock numbers and genotypes are; BL-4084: *eve*[*5*]/*SM6a*, BL-5344: *eve* [*1*]/*CyO; P{ry[+t7.2]=ftz/lacC}*, BL-1719: *Df(2R)X3/CyO, Adh[nB]* and 1702: *Df(2R)X1, Mef2[X1]/CyO, Adh[nB]*. Three virgins of a mutant stock were crossed with 3 males from each of the 20 inbred lines, and allowed to lay eggs for 2–3 days. The offspring were counted and sexed, between 10 and 11 am, from day 10 to 18. The experiment was fully balanced and repeated three times, several weeks apart. The parents of all lines used in the crosses had been grown for 2 generations under controlled density (parents discarded between days 2 and 5 depending on visual assessment of egg number). We recorded both the total number of offspring (viability), and developmental time, summarized as the average time to eclosion for a given combination of, mutation, cross, genotype, sex and replicate.

For the association tests, 60 inbred lines where studied. The *Hb8Δ/wt* polymorphism was genotyped in three individuals of each line in the generation that was phenotyped. The rearing and measuring procedure was identical to the first experiment, except no crosses were required and only replicates were measured (two weeks apart).

Embryo Collections, Fixing and Staining

The embryos were collected, fixed and stained with standard protocols, as we have done before [8]. Four inbred lines with (NC25 and NC128) and without (NC006, NC017) the *Hb8Δ* laid eggs for 4–5 hrs at 22°C. Briefly, we collected embryos from each of the four lines, and they were fixed. Multiple embryo collections were pooled before staining with Eve primary antibody and a secondary antibody. The histochemical LacZ staining reaction was run for 12 minutes. The stained embryos were stored in 70% glycerol at 4°C, and photographed within a week.

Photography and Measurements

Each embryo in the appropriate developmental stage range was photographed three times at 20X magnification with water immersion on a Zeiss microscope. First a DIC sagittal section yielding maximum length of embryo and then two sections (DIC sagittal and bright-field) captured the stripes. Tiff photographs were saved and the X and Y coordinates of stripe boundaries assessed in ImageJ (rsb.info.nih.gov/ij/ [57]). First, a straight line was superimposed on the sagittal image, and the X-Y coordinates of anterior and posterior of the embryo recorded. Second, the same guideline was superimposed on the other two images and X-Y landmarks of the anterior and posterior boundary of each stripe were visually assessed and recorded. Third, the rotation of the embryo along the Dorsal/Ventral axis was scored. Finally, the stage of development was also visually assessed from *eve* pair-rule expression, in increments of 0.5 on the scale from 1 to 5, around cellularization [11]. The same investigator (AP) did all measurements.

Summarizing the Expression Pattern

The raw landmark data indicating the length of the embryo and placement of stripes were processed in two ways. The relative positions of stripe boundaries were estimated by calculating distance of landmarks from the anterior and posterior end using standard geometric formula. First, the length of the embryos was estimated. Second, the relative distance from one embryo tip to the anterior and posterior boundary of each stripe was calculated.

Statistical Analysis of Adult and Embryonic Phenotypes

SAS version 8.2 [58] was used for analyses of phenotypes. The viability and developmental time analyses were conducted with mixed model ANOVA (proc MIXED). The model for the test-cross was:

$$Y = M + C + MXC + G + MXG + CXG + S + O + L(CXG) + error$$

Denoting the fixed effects of the mutation (M), that is the 4 different eve deficiencies or point mutations, the cross (C) designating the balancer (CyO) or the "loss of function" (LoF) *eve* mutation, the genotype (G) term which evaluates the effects of *Hb8Δ*, sex (S) and appropriate interaction terms. The effects of Line (L) and replicate vials (R) are considered random factors. Furthermore, the total number of offspring (O) was included as a covariate. As a large factorial model with 4 fixed terms runs the risk of being overly parametrized, higher order terms were

evaluated and dropped if they were not significant at the 0.05 level. The association tests of the inbred lines data were simpler, with only terms denoting genotype, sex and total number of offspring, and not described here.

The relative location of histochemically detected Eve stripes was studied similarly. In order to remove the effects of orientation, a reduced model was fit, and the residuals were used in the subsequent analysis. The positioning of stripes was analyzed with a mixed model ANOVA. The dependent variables of interest are the relative positioning of stripe boundaries, with the anterior boundary of stripe 3 (S3A) and the posterior position of stripe 7 (S7P) being particular candidates given prior evidence on Hunchback distribution in the embryo [59]. The ANOVA model had the general form:

$$Y = G + T + GXT + L(G) + error$$

Where G, indicating genotype (the presence or absence of Hb8), is a fixed main effect. The covariate T (for developmental time) captures the developmental progression and L is a random term for different inbred lines. The relative stripe position matrix (anterior/posterior boundary of all 7 stripes) was also summarized with Principle component (Proc PRINCOMP) on the correlation matrix. Only the first component, with eigenvalue 7.42, was analyzed for dependence on Hb8 genotype.

Results

Polymorphism in Regulatory DNA Includes Large Deletions of TFBS

First we surveyed the molecular variation, *i.e.* nature, frequency and distribution of polymorphisms, in 13 well studied *Drosophila* regulatory elements and several less well defined elements and spacer sequences. Few indel polymorphisms are found in the regulatory regions, 8 of the regions have no indels (Table 1). Purifying selection seems to affect both SNP and indel polymorphism, as there is a significant correlation between θ for SNPs and indels ($r = 0.48$, $p = 0.03$, Figure S1A). The size and frequency of indels in characterized *cis*-elements was contrasted to those in non-coding regions surrounding two developmental genes, *hairy* and *EGFR* [46,60]. As was previously observed [46] most indels are short, and rarely do large indels (more than 10 bp) reach appreciable frequency (Figure S1B). The notable exception is a 72 bp deletion in the stripe 3 and 7 enhancer (s3+7e) of *eve* (Figure 1A and B). Interestingly this deletion removes a DNase I characterized Hunchback (Hb) binding site [55], and is henceforth called *Hb8Δ*. Bioinformatic analyses in Jaspar show that this site has a PWM score of 8.5, suggesting the notion that this a transcription factor binding site presence/absence polymorphism. Oddly enough, less than 250 bp away (in the spacer separating *s3+7e* and *s2e*), another segregating large deletion also removes a putative Hunchback binding site (Figure 1C). This site (here called Hbs1) is predicted with high PWM score, 11.2. That is the fourth highest score of 60 predicted Hb sites in the 8 kb region upstream of *eve* in *D. melanogaster* (Figure S2A and Table S2). Most of the 21 DNaseI characterized Hb sites in *s3+7e* and *s2e* have lower scores than Hbs1. This 45 bp deletion in the spacer is referred to as *Hbs1Δ*. This putative Hb binding site has probably been unnoticed for two reasons. It sits outside the fragments tested for enhancer function, presumably because of restriction site locations [10,37]. Also, the *D. melanogaster* reference genome sequence contains the deletion. To iterate, the 45 deleted bases do not appear in the standard versions of the *D. melanogaster* genome and are only visible in genomic alignments with close *Drosophila* relatives or population genetic sequence data. The two deletions sit on distinct haplotypes, and are never found in the same inbred lines. They are both at appreciable frequency, in a sample of 55 Costa Rican chromosomes the *Hb8Δ* and *Hbs1Δ* are at 9% and 17% frequency respectively (Figure 1B and C). This leads to the question, are these deletions harmful, neutral or beneficial?

Phylogenetic Footprinting of s3+7e shows the Hb Sites are Conserved

Comparative genomic alignments of the *s3+7e* and the adjacent regions with 12 publicly- available *Drosophila* genomes [61] were used to assess the functional importance of these two predicted Hb binding sites, and other characterized Hb, Kni and Stat sites [32,55,59]. Similarly to the *eve s2e*, TFBS in s3+7e are highly conserved (Table S3); 3 of 13 Hb sites are identical from *D. melanogaster* to *D. mojavensis* and 9 have none or only one mutation between *D. melanogaster* and *D. persimilis*. The Hb8 site is found in all of the 12 species, except *D. ananassae* (most probably due to a gap in the genomic sequence), but has experienced several substitutions (Figure 1D). The PWM score for Hb8 is 8.2 in *D. melanogaster* and *D. simulans*, but 9.9 in *D. yakuba* and *D. pseudoobscura* (Figure S2 and table S2). On the other hand, the predicted Hbs1 site (with a PWM score of 11.2) is completely conserved between *D. melanogaster* and *D. yakuba*, but was not found in distantly related species. Those data suggest considerable evolutionary constraints on those sequences, arguing that they could indeed be functional Hb binding sites. But in the absence of functional tests they must regarded as putative Hb binding sites.

Additionally, the *Hb8Δ* also removes half of a putative Sloppy Paired 1 (Slp1) binding site. The putative Slp1 site is less conserved then the characterized Slp1 site in *s2e* [62] (Table S4), but no SNPs within either of these two (characterized and putative) Slp1 binding sites in *eve*, in 104 sequenced alleles, suggests selective constraint within *D. melanogaster* at least. The genome comparisons confirm that both Hb binding sites in *eve* affected by these two deletions have been protected by purifying selection. This prompts the question, why do these deletions of conserved TFBS occur at such high frequencies in populations? Here we focus mainly on studying the population genetics of *Hb8Δ* and assess its potential impact on development and fitness.

Polymorphism on the *Hb8Δ* and *wt* Haplotypes

How can a deletion removing a conserved binding site be at such high frequency in the population? One possibility is that the deletion of Hb8 is buffered by compensatory mutations (sitting on the same haplotype). To assess this, and to evaluate the polymorphism in the region, two strategies were deployed. One was deeper sequencing of four *eve* regions (the promoter, *s2e, s37e* and the *late element*) in inbred strains from North Carolina, and the other, a contrast of sequence diversity in alleles with or without the *Hb8Δ* in ~8 kb around *s3+7e*.

The *Hb8Δ* is at 32% frequency in the NC population (N = 63), and there is less variation on the *Hb8Δ* haplotypes compared to the *wt* haplotypes (Table 2). For instance π (which captures the number of substitutions and their frequency) is 25% to 100% lower on the *Hb8Δ* haplotypes. This is most extreme in the *s3+7e*, and notably weaker in flanking regions. This tendency was captured by other population genetic summary statistics (S, Haplotypes, haplotype diversity and Dxy – a measure of differences in nucleotide substitution rate between samples). Furthermore, no unique mutations are found on the eve-*Hb8Δ* haplotypes; the variation observed on the *Hb8Δ* haplotypes is all presumed to be due to recombination. These observations suggest

A

B

C

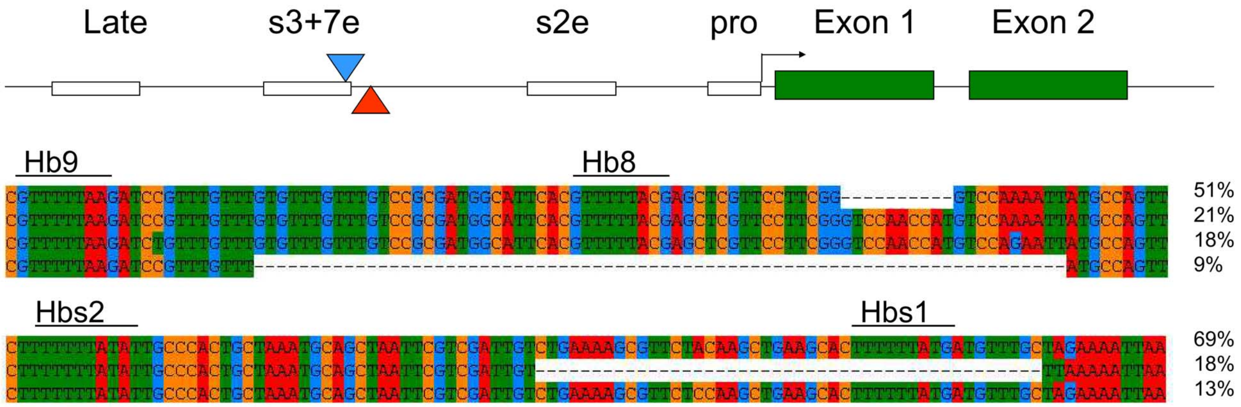

D

	Hb14a	Kni5	Stat2	Hb10	Hb8	Hbs1
D.mel	TTTTTTGTTT	CTGCGCTAGTT	TTCCCCGAA	TTTTTTAATTC	GTTTTTACGA	TTTTTTATGA
D.sim	....	....	....	....	....	....
D.sec	....	........T..	....G....	....	....	....
D.yak	....	......C.C..	....	....	A......T..	....
D.ere	.........T	......C....	....	....	A......T..	....
D.ana	....	....	.....A...	....	N/A	N/A
D.pse	....	....	....	....	A......T..	N/A
D.per	....	....	....	....	A......T..	N/A
D.vir	N/A	....	....	....	A......TT.	N/A
D.gri	N/A	....C......	....	....	A......TT.	N/A
D.moj	N/A	....	....	....	A......TT.	N/A

Figure 1. Two large deletions remove conserved Hunchback binding sites in the *eve* stripe 3+7 enhancer. A) The structure of the upstream region of *eve*, open boxes represent the late element, *s3+7e*, *s2e* and promoter regions, and green boxes the two exons. The deletions are shown by blue (*Hb8Δ*) and red (*Hbs1Δ*) triangles. B) Detailed structure of the *Hb8Δ* and frequency of the four alleles at this position in a Costa Rican population. C) Structure of *Hbs1Δ* and frequency of alleles in the same population. D) The conservation of a subset of TFBS in the *s3+7e* and the Hbs1 site. Full species names are provided in Materials and Methods and data for other *s3+7e* binding sites in Table S3.
doi:10.1371/journal.pone.0091924.g001

positive selection favors the *Hb8Δ* or linked variants. However none of the standard population genetics tests (Tajima's D or Fu and Li's statistics) indicate positive selection (data not shown); neither did the Hudson *et al.* (1993) haplotype test ($p > 0.73$ for each of the four regions).

We next compared more extensively the sequence variation on the *Hb8Δ* and *wt* chromosomes and screened for variants that might possibly compensate for the loss of this Hb binding site. We estimated the polymorphism on two distinct haplotypes carrying either the *wt* or deletion polymorphism, by sequencing 16 (*Hb8Δ*) and 18 (*wt*) chromosomes of each type. The 8200 bp region we selected spans the *eve* neighborhood, from the 3′UTR of CG12134 to the end of the transcript. There is reduced polymorphism (π and θ) on the *Hb8Δ* haplotypes compared to *wt* haplotypes (Figure 2A and B), which is consistent with selection for the *Hb8Δ* bearing haplotype. Another indicator of long haplotypes is high LD between *Hb8Δ* and polymorphic sites in the region (Figure 2C). Several sites more than 3 kb away from Hb8 are in high LD ($r^2 > 0.7$) with the deletion. Additionally, most polymorphism in the region shows perfect coupling or repulsion LD to *Hb8Δ* (data not shown). (The *Hbs1Δ* was only found in 3 (*wt*) lines. Omission of those 3 lines did not affect the outcome of the polymorphism analyses - data not shown). Furthermore, no variants are unique to the *Hb8Δ* haplotype. Finally, no potential compensatory mutations that strengthened or generated other Hb sites were observed. The data do reveal less diversity on the *Hb8Δ* haplotype, compared to the *wt* haplotype. Note however, standard tests of natural selection can not be deployed on these data because the sampling was not random from a population; lines were picked for sequencing to get similar representation of *wt* and *Hb8Δ* chromosomes.

Geographic Distribution of the *Hb8Δ*

What is the geographic distribution of *Hb8Δ* and does it correlate with geographic attributes? To study the geographic distribution, bulk DNA samples from 51 cosmopolitan samples, from Europe, Africa, Asia and South America [44] were genotyped with allele specific primers. There was evidence of *Hb8Δ* in 43 of the 51 populations (Table S5), consistent with an evolutionarily old and broadly distributed polymorphism. The cosmopolitan distribution of the *Hb8Δ* is unlikely if it was strongly deleterious.

Does this binding site deletion show any relationship with geographic attributes? To assess this we genotyped *Hb8Δ* in 13 east coast samples, from Maine to Florida [43]. The frequency ranged from 5% to 35% (Table 3) but there was not a significant relation between latitude and frequency of *Hb8Δ* ($b = -0.006$, $p = 0.1$). For comparison the *s2e* was also sequenced in the same individuals. Again, no unique SNPs are found on the *Hb8Δ* haplotype. Thus, nothing in in this broader N-American sample

Table 1. Single base and indel polymorphism in *D. melanogaster* regulatory elements.

			SNPs				Indels			
**Gene**	**region**	**Sites**	**S**	π	θ	**Tajima's D**	**S**	π	θ	**Tajima's D**
Antp	promoter	512	9	0.0037	0.0055	−1.24	2	0.0006	0.0010	−1.20
salm	wing blade enhancer	432	12	0.0088	0.0090	−0.05	0			
en	regulatory element	554	6	0.0031	0.0034	−0.36	0			
Ubx	Bxd promoter	346	1	0.0008	0.0009	−0.29	0			
ems	Abdominal enhancer	604	10	0.0043	0.0052	−0.68	0			
Hb	promoter spacer[#]	184	0				0			
tll	promoter	399	9	0.0070	0.0071	−0.05	0			
Act57B	promoter	489	12	0.0082	0.0077	0.24	2	0.0007	0.0013	−1.28
RpL29/CG30390	promoter	482	19	0.0108	0.0124	−0.53	4	0.0009	0.0026	−2.19
RpL30	promoter	563	9	0.0053	0.0048	0.35	0			
Kr	promoter	492	6	0.0053	0.0037	1.47	0			
Kr	CD1(a)*	444	20	0.0138	0.0121	0.53	1	0.0002	0.0006	−1.16
Kr	CD1(b)*	334	22	0.0124	0.0181	−1.17	2	0.0006	0.0016	−1.39
eve	promoter	373	6	0.0043	0.0050	−0.44	0			
eve	promoter spacer[#]	545	6	0.0036	0.0029	0.79	2	0.0003	0.0010	−1.40
eve	*s2e*	593	11	0.0051	0.0041	0.67	1	0.0001	0.0004	−0.87
eve	*s3+7e*	283	3	0.0024	0.0025	−0.05	2	0.0012	0.0016	−0.49
eve	*s3+7e* spacer[#]	432	12	0.0088	0.0065	1.07	4	0.0009	0.0022	−1.41
eve	Late element	386	8	0.0040	0.0046	−0.36	0			

*Kr CD1 region was sequenced in two parts – and is presented as such due to incomplete genotyping.

[#]The region just upstream of the *eve* and *hb* promoters are called "promoter spacer", and similarly the region proximal of *s3+7e*.

doi:10.1371/journal.pone.0091924.t001

Table 2. Polymorphism in four regulatory elements of *eve* among inbred lines from North Carolina.

Region	Length	Sample*	S	π	Dxy	Haplotypes	Hd
Late	327	All	4	0.0034	0.0036	7	0.8
		wt	4	0.0034		6	0.748
		Hb8Δ	3	0.0028		5	0.663
s3+7e	262	All	6	0.0103	0.0131	7	0.805
		wt	6	0.0098		7	0.8
		Hb8Δ	0	0		1	0
s2e	547	All	11	0.0050	0.0057	18	0.859
		wt	11	0.0048		13	0.862
		Hb8Δ	8	0.0021		6	0.447
Pro	565	All	12	0.0052	0.0054	15	0.864
		wt	11	0.0056		12	0.863
		Hb8Δ	7	0.0020		5	0.442

*Sample size: All (N = 63), *wt* (N = 43), *Hb8Δ* (N = 20).
S: segregating sites.
Dxy: Average number of nucleotide substitutions per site between *wt* and *Hb8Δ* samples.
Hd: Haplotype diversity.
Pro: Promoter.
doi:10.1371/journal.pone.0091924.t002

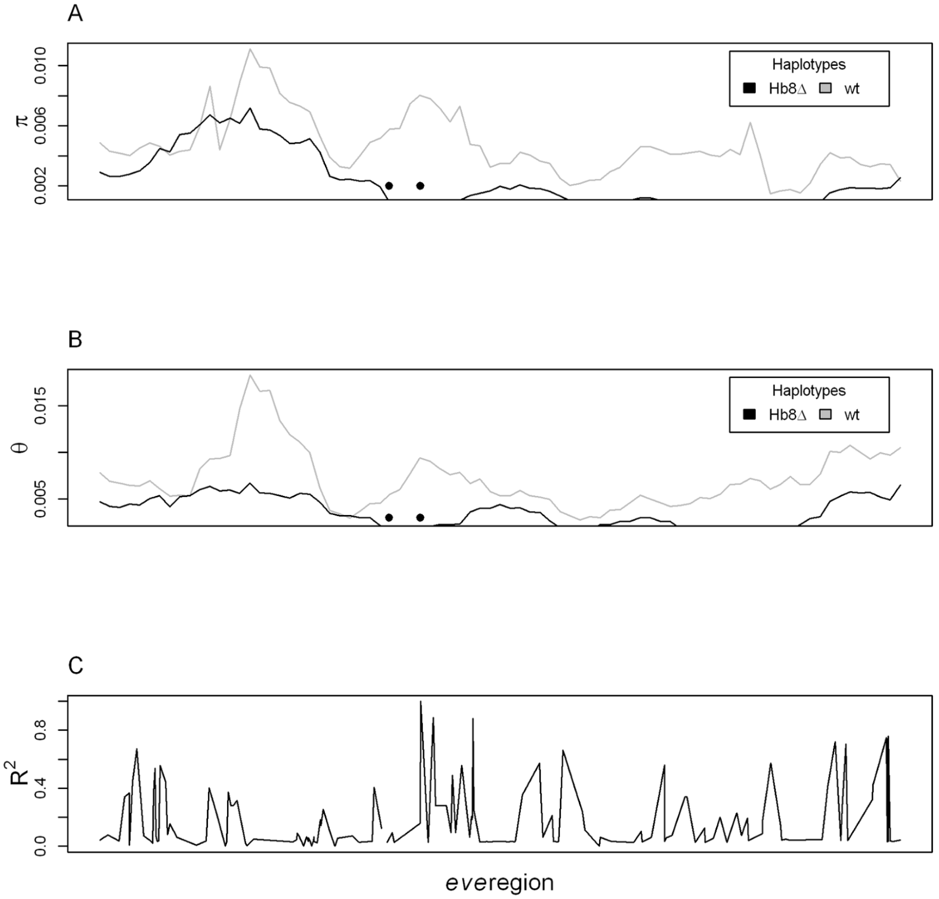

Figure 2. Polymorphism in the ~8200 bp *eve* region. Visualized are positions 5,860,182–5,868,302 on 2R, with the *Hb8Δ* at position 3292 and *Hbs1Δ* at 3602 (black dots). Contrast of polymorphism in the *Hb8Δ* (black) and *wt* haplotypes (gray), with π in A) and θ in B), in 800 bp windows, sliding 100 bp. C) LD between the *Hb8Δ* and other variant in the region, estimated with r^2.
doi:10.1371/journal.pone.0091924.g002

suggests complementary mutations in *s2e*. Curiously however, there is a significant reduction in *s2e* polymorphism with latitude ($p = 0.02$ for π and θ). This does not explain the prevalence of *Hb8Δ*, but suggests geography (or history) affects variation in the regulatory regions of some developmental genes.

Testing for Effects of *Hb8Δ* on Viability and Developmental Time

Test crosses and analysis of inbred lines were used to gauge the putative impact of *Hb8Δ* on the number of offspring hatching and developmental time. Here developmental time is assessed as the time to eclosion (see methods).

Consistently with earlier studies [63,64] hemizygosity at *eve* reduces viability (Table 4) by about 20% in all crosses except to *eve*^{5} (DF vs. Cy in Figure S3). However offspring number was not affected by the deletion of Hb8 binding site (Genotype term in Table 4). Number of hatching offspring differs between the four *eve* mutant stocks (Table 4) most likely due to varying genetic backgrounds. We also asked about factors influencing developmental time. The ANOVA's indicate difference among *eve* alleles, and potential effects of hemizygosity at the locus (Table 4). Most notably, *Hb8Δ* seems to reduce developmental time (Table 4) – while hemizygosity at *eve* increases it. In three of the four crosses did *Hb8Δ* individuals develop significantly faster than the *wt* flies (Figure 3). The *Hb8Δ* flies eclose on average 3.5 hours earlier, but again no effects are seen in *eve*^{5}. This effect was also seen if the effect is estimated for sexes separately. In 13 of the 16 Mutation-Cross-Sex combinations *Hb8Δ* developed faster than flies with *wt s3+7e*, which is significant in a sign-test (binomial, $p = 0.02$). Note the *Hb8Δ* is tested in heterozygous form, thus in these crosses it appears to have dominant effects on developmental time.

We also examined the effects of *Hb8Δ* with association tests in 60 inbred lines. As before, *Hb8Δ* had no effect on offspring number. Peculiarly, the data do not confirm the association between *Hb8Δ* and developmental time (lower part of Table 4). The estimated developmental time is in the same range for both experiments suggesting they are not systematically different. Together these data suggest an effect of *Hb8Δ* on developmental time, but further tests are needed to confirm or refute this.

Histochemical Staining of Eve Expression

Proximal phenotypes, like protein level at a specific time and location in the embryo, might be associated with functional variation in regulatory elements. To test this we stained for Eve in stage 14A embryos of four inbred lines, two *Hb8Δ* and two *wt*. Mixed model ANOVA shows that the relative positioning of the Eve stripe boundaries differs between the four inbred lines studied (Table S6). Both developmental stage and embryo orientation affect the anterior and posterior boundaries of stripes. Those sources of error were accounted for by i) working with the residuals after fitting the embryo orientation and ii) using developmental stage as a covariate. The average developmental stage does not

Table 3. Frequency of *Hb8Δ* and *s2e* polymorphism along the east coast of North America.

Populations		*Hb8Δ*		*s2e*				
**Location, State**	**Latitude**	***Freq.***	F_{ST}	**N**	**Sites**	π	θ	F_{ST}
Homestead, FL	25° 2′	0.32		24	5	0.0020	0.0024	
Merrit Island, FL	28° 3′	0.16	0.051	26	7	0.0024	0.0033	0
Jacksonville, FL	30° 2′	0.19	0.000	29	7	0.0022	0.0032	0
Eutawville, SC	33° 2′	0.20	0.000	26	5	0.0021	0.0024	0
Smithfield, NC	35° 3′	0.14	0.000	16	4	0.0024	0.0022	0
Richmond, VA	37° 3′	0.05	0.033	33	5	0.0018	0.0022	0
Churchville, MD	39° 3′	0.17	0.052	23	5	0.0018	0.0024	0.051
Middlefield, CT	41° 3′	0.09	0.017	37	5	0.0019	0.0022	0.007
Concord, MA	42° 0′	0.19	0.030	41	5	0.0014	0.0021	0.009
Whiting, VT	43° 6′	0.17	0.000	30	2	0.0015	0.0010	0
All		0.17	0.055(0.04)*	285	11	0.0020	0.0032	0.029(0.02)*

The *s2e* amplicon was 555 bp.
Sample size for *Hb8Δ* was 380.
*Average F_{ST} (standard deviation). None of these pairwise F_{ST} are significant after Bonferroni correction.
doi:10.1371/journal.pone.0091924.t003

Table 4. ANOVAs testing for the effect of *Hb8Δ* (genotype) on viability and developmental time.

	Viability				Developmental time			
Exp[a]	Term/ Var.Comp	df	F/ Est(SE)[b]	*P*	Term/ Var.Comp	df	F/ Est(SE)[b]	*P*
Test Cross	Mutation	3,493	55.15	9.4E−31	Mutation	3,486	5.52	9.8E-04
	Cross	1,36	20.06	7.3E-05	Cross	1,36	0.46	5.0E-01
	M X C	3,493	6.81	1.7E-04	M X C	3,486	2.64	4.9E-02
	Genotype	1,36	0.09	0.77	Genotype	1,36	12.62	1.1E-03
	M X G	3,493	6.39	3.0E-04	M X G	3,486	1.46	0.22
	C X G	1,36	0	1.00	C X G	1,36	0.28	0.60
	Sex	1,493	1.04	0.31	Sex	1,486	6.67	0.01
	$V_{Line(CG)}$[c]		10.8(4.1)	3.9E-03	Offspring	1,486	4.53	0.03
	V_{error}[c]		80.9(5.2)	9.3E-56	$V_{Line(CG)}$[c]		25.5(15.1)	0.05
					V_{error}[c]		538.7(34.4)	1.9E-55
Inbred lines	Genotype	2,53	0.26	0.77	Genotype	2,53	1.71	0.19
	Sex	1,136	3.93	0.05	Sex	1,132	3.4	0.07
	G*S	2,136	0.26	0.77	G*S	2,132	0.73	0.48
	$V_{Line(G)}$[c]		109.2(26.4)	1.8E-05	Offspring	1,132	0.02	0.90
	$V_{Rep(L)}$[c]		27.2(8.6)	8.0E-04	$V_{Line(G)}$[c]		231.6(63.4)	1.3E-04
	V_{error}[c]		53.1(6.4)	8.2E-17	$V_{Rep(L)}$[c]		122.4(29)	1.2E-05
					V_{error}[c]		107.1(13.6)	2.0E-15

Mutation tests for differences among *eve* allele stocks, Cross the balancer vs loss-of-function *eve* allele, and genotype the *wt* vs. *Hb8Δ*.
[a]Experiment: a test cross of 20 lines with defined genotype to four *eve* mutants and genotype tests on the 60 inbred lines.
[b]For fixed terms the F-statistic is reported and for the random terms the estimated variance components (e.g. $V_{Line(C\ G)}$) with standard error.
[c]The significance of the variance components was determined by the *z*-function. The variance component for Developmental time was multiplied by 1000 for representation.
doi:10.1371/journal.pone.0091924.t004

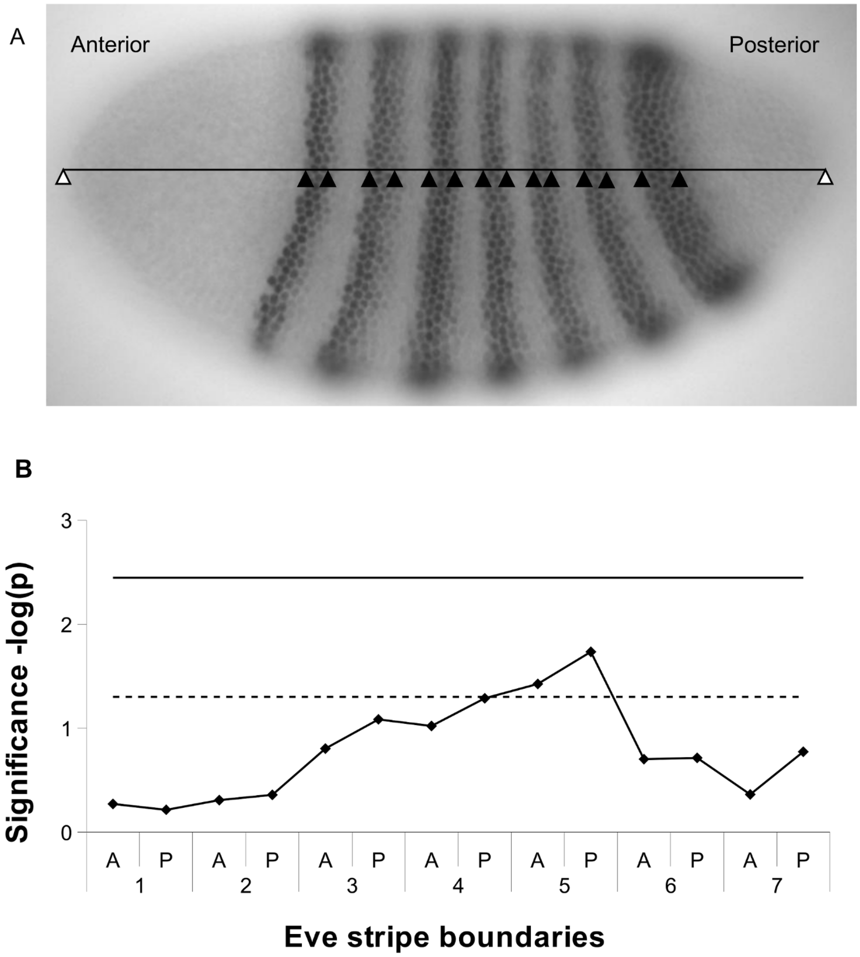

Figure 3. Effects of *Hb8Δ* on developmental time. Represented are least square mean estimates for combination of eve mutation (alleles and deficiency chromosomes), and balancer (Cy) or mutation carrying chromosome (DF). Error bars represent 95% confidence intervals. Developmental time was estimated as the time to eclosion, see methods.
doi:10.1371/journal.pone.0091924.g003

differ between lines (p = 0.8), suggesting that rate of early development does not contribute to the line differences.

Hb repression establishes the anterior boundary of stripe 3 and posterior boundary of stripe 7 [62]. Thus, *a priori*, those features are most likely to be affected by *Hb8Δ*. However, the mixed model ANOVA does not indicate effects of the *Hb8Δ* on these stripe 3 and 7 boundaries (Figure 4). It is possible that this Hb site has broader function. The only putative signal in the data was with stripe 5; according to least square means stripe 5 is found more anteriorly in *Hb8Δ*. But this is not formally significant after Bonferroni correction for all 14 tests. A complementary analysis of principle components (PC) of the relative stripe positions does not implicate *Hb8Δ* in stripe positioning. The two largest principle components capture variation in (PC1) the central stripes and (PC2) the anterior – posterior axis of the embryo. The contribution of *Hb8Δ* to principle component 1 is not formally significant ($F_{1,10} = 4.25$, $p = 0.07$). These results do not suggest that *Hb8Δ* affects Eve pattern in the early development.

Discussion

Sequence comparisons of close and more distantly related species show how TFBS emerge, change and get lost [8,65]. Is this turnover of functional sequences due to relaxed purifying selection, or does positive selection play a role [66–68]? There is substantial variation in gene expression among individuals and the bulk of expression QTLs map in *cis* [69–71]. The exact nature of those *cis* variants is rarely known, but a systematic review by Rockman and Wray [72] shows that SNPs, indels and length polymorphism in repeats can abolish TF binding and affect expression of neighboring genes.

Hunchback Site Polymorphisms are not Deleterious

Here we report that two large deletions segregating at moderate frequency remove predicted Hunchback binding sites in, and next to, the stripe 3 and 7 enhancer of *eve*. Both sites have high PWM scores and are evolutionarily conserved. One of them (Hb8) was characterized molecularly [55]. Three observations suggest that Hbs1, removed by a 45 bp deletion, is a true Hb binding site. It has among the highest PWM score of Hb sites in the *eve* region. It is evolutionarily conserved between *D. melanogaster* and *D. erecta* and resides less than 250 bp away from the Hb8 site. Stanojevic *et al.* [55] footprinted 4 Hb sites in the spacer between *s2e* and *s3+7e*, and recent thermodynamic models and quantitative measurements of TF abundances indicate that the spacer between *s2e* and *s3+7e* contains functional Hb motifs [73]. However functional assays are required to confirm that Hb binds to these two sites *in vivo* and modulates *eve* expression.

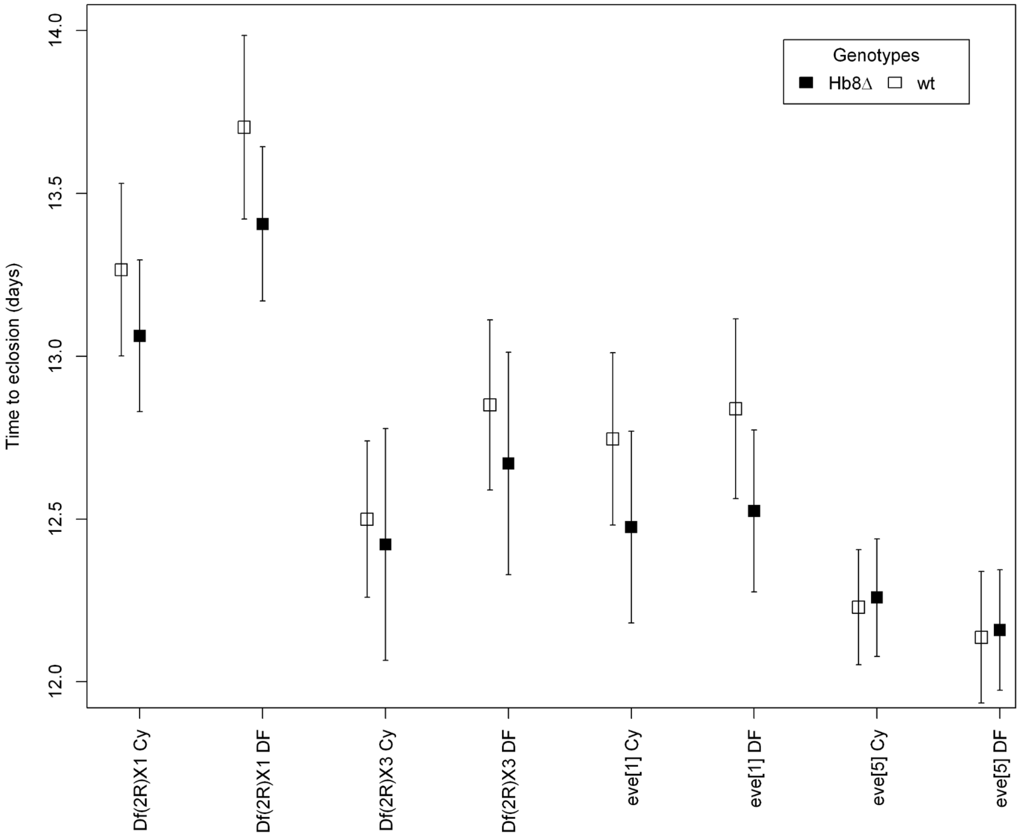

Figure 4. Testing for effects of *Hb8Δ* on Eve stripes. A) Measurement of *eve* stripe positioning. A surface image is used for measurement of stripe boundaries. A line was superimposed on the embryo and stripe boundaries visually assessed and recorded as X-Y coordinates (black triangles). Coordinates for embryo ends (white triangles) are measured from sagittal slices (not shown). B) Significance (negative log of p for genotype; *Hb8Δ* vs. *wt*) along the embryo. Shown are lines corresponding to the -log ($p = 0.05$) cutoff (dashed line) and the Bonferroni correction for 14 tests -log($p = 0.0035$) (solid line).
doi:10.1371/journal.pone.0091924.g004

Our initial hypothesis was that these deletions of Hb binding sites are deleterious, as the loss or modulation of a single TFBS can have measurable effects [72,74,75]. This is refuted by several facts: 1) both mutations are at appreciable frequency, 2) individuals homozygous for each of those deletions survive as inbred stocks, 3) *Hb8Δ* has cosmopolitan distribution and 4) *Hb8Δ* does not seem to reduce viability and, if anything, it speeds up developmental time. The genetic assays had sufficient statistical power to detect the effects of *eve* hemizygosity on offspring number (consistent with reported partial haplo-insufficiency at the locus [63,64]) and less so developmental time. Thus we conclude that the *Hb8Δ* is not strongly deleterious. The alternate scenarios are that the two deletions are either (nearly) neutral or favored by positive selection.

The most parsimonious explanation is that *Hb8Δ* is neutral and drifts in the population. This scenario is supported by haplotype tests, which do not point to the involvement of positive selection. However, the fact that the two deletions destroy binding sites for the same TF in the same enhancer is rather puzzling. Thus, it is tempting to hypothesize that the two Hb binding site deletions are favored by selection. Curiously, no other Hb sites in the *s3+7e* or *s2e* are affected, no substitutions are seen in more than 100 sequenced lines.

Variation in Early Development

Several studies have documented substantial variation in early Drosophila gene expression, with expression arrays [76], RNA seq [77] and *in-situs* [78]. As the deletions are found in *s3+7e,* it is most probable that they could affect Eve stripes 3 or 7. Hb is abundant in the anterior of the embryo, and drops adjacent to the anterior boundary of *eve* stripe 3. Hb is also produced in a narrower domain in the posterior, close to the posterior boundary of *eve* stripe 7 [62]. Hb demarcates the boundaries of those stripes (and stripes 4 and 6). Thus deletions of Hb sites would be expected to lead to an anterior shift of stripe 3 and posterior shift of stripe 7, because this regulatory module would be less sensitive to Hb repression (the absence of its full complement of binding sites). Our analysis of Eve expression in four inbred lines does not reveal effects of *Hb8Δ* on Eve stripe placement. Genetic and maternal factors affect the placement of expression boundaries; physical or environmental attributes like egg size do as well [78–80]. Note, lack of evidence does not prove the alternative. These results do not prove that the *Hb8Δ* does not affect Eve expression. The ideal test of the functionality of *Hb8Δ* and *Hbs1Δ* requires transgenic constructs in a common genetic background or homologous recombination into the *eve* locus of a particular line. It is unclear how such alterations would affect proximal or distal features of development. The quantitative tests suggest *Hb8Δ* acts dominantly, and speeds up development by ~ 3 hours. This seems unrealistic as the Eve pair rule pattern only takes ~50 minutes to mature [81], thus it is impossible that these effects (if real) are due to Hb and *eve* interaction during early development. But curiously both *eve* and *hb* also play a role in the developing neuronal system [82,83] but

the functional interaction of Hb and *eve* in those tissues is largely unexplored. In the absence of functional or genetic confirmation we argue for cautious interpretation of the observed association of *Hb8Δ* and developmental time in the test-crosses. Finally, it is also possible that these deletions affect proximal developmental events, but that those effects are a minute or acceptable noise in the system.

Can Co-evolution Explain the High Frequency Hb TFBS Deletions?

Co-evolution can occur via neutral changes (e.g. in the network neighborhood [84]) or via positive selection favoring compensatory changes in the genome. Here two co-evolution models that may account for these two Hb binding site deletions in *eve* are entertained. Those are i) *cis*-changes within *eve* or, ii) *trans*-changes in the function or abundance of activators and/or repressors.

First, the relatively high frequency of those two deletions could reflect co-evolution within *eve*. Hunchback acts both as a transcriptional activator and repressor during development [85–87]. Hb positively influences expression via the *eve* stripe 2 enhancer, but is part of two-tier repressor system that demarcates the boundaries of stripes 3, 4, 6 and 7 [62]. Stripes 3 and 7 are known to be activated by D-stat [32], an ubiquitously available activator (other agents may also play a role). The high frequency of Hb binding site deletions could be a co-evolutionary response to increased activation of stripes 3 and 7 expression, for instance via altered Dstat binding. This is unlikely as the two D-stat sites in *s3+7e* have not diverged between *D. melanogaster* and *D. erecta* (Table S2) and no polymorphism is found in those sites within *D. melanogaster*. Binding sites for other agents activating *eve* stripes 3 and 7 may have changed; TFBS that could reside elsewhere in regulatory regions around *eve*. The *eve* regulatory region is 85–95% identical between *D. melanogaster* and *D. simulans*. We scanned the *eve* region of both species with Jaspar [56], and found hundreds of TFBS differing between the species (data not shown). Nonetheless, no changes in Hb or Dstat sites were found. It is also possible that miRNA docking sites or other regulatory elements in *eve* have changed, thus leading to selection for higher frequency of those two Hb site deletions.

Alternatively, changes in structure or function of *trans*-factors, like Hb itself, may have led to the increased frequency of those two Hb binding site deletions. It is improbable that a protein change is responsible, as the differences between the *D. melanogaster* and *D. simulans* Hb proteins are all on the *D. simulans* branch (unpublished results, Dagmar Yr Arnardottir and Arnar Palsson). We find it more plausible that the spatial or temporal amount of *trans*-factors has changed, for instance a lower amount of Dstat. The most intuitive scenario is, quantitative, temporal or even spatial changes in Hb expression in the embryo – which may have prompted co-evolution in regulatory elements sensitive to quantitative changes in Hb amount in development. The *eve s3+7e* might be such a critical Hb-target element. This is of course speculation, but in this scenario, one would expect that other Hb target enhancers, which produce expression overlapping the spatial and temporal patterns of *eve s3+7e* might also have experienced altered selection pressure. Thus, other Hb such target genes could also exhibit point mutations or deletions of conserved and presumably functional Hb binding sites. Note, we are not arguing positive selection is necessarily responsible; changes in Hb dose could lead to relaxation of selection for a subset of Hb target genes, and thus previously detrimental mutations in these genes could drift to higher frequency.

Conclusions

The genetic network governing early *Drosophila* development has been used to discover many of the basic principles of developmental genetics, regulatory DNA function and regulatory evolution [6,10,88,89]. Recent technical and analytical improvements have enabled quantitative analyses of enhancer function and logic [87,88,90–92] and dosage compensation [77,93]. Developmental networks must cope with variation due to chance, the internal and external environment, and in the relevant genetic components. Studies point to the involvement of positive selection in the gain and loss of TFBS in *Drosophila* [66,94] and co-evolution within enhancers [39,95]. Furthermore, non-clocklike evolution of the *s2e* from four *Drosophila* species [11], indicates co-evolution of TF abundance and functional elements in *cis*-regulatory modules. The fact that two large deletions removing TFBS for Hb are found in close proximity in a regulatory element, might be an example of such co-evolution. However we favor the cautious explanation that these high frequency deletions reflect developmental system drift [40,41], i.e. permitted deviations in parameters of the *Drosophila* developmental regulatory network.

Supporting Information

Figure S1 Constraints on SNPs and indels in regulatory DNA. A) The relationship between single base and indel polymorphism (summarized with θ) in 19 enhancers and promoters in *D. melanogaster*. Many of the characterized enhancers have no indels, and sit therefore at $Y = 0$. B) Size and frequency of indels in characterized regulatory DNA and proximate promoters (dark circles) vs. indels in non-coding regions (open circles) around two developmental genes (*hairy* and *EGFR*).
(TIFF)

Figure S2 Comparative genomics of predicted Hb binding sites in *eve*. The strength (height of bar) and location of Hb binding sites predicted with JASPAR in the ~8 kb region upstream of *eve* transcription start site, in four *Drosophila* species, A) *D. melanogaster*, B) *D. sechellia*, C) *D. yakuba* and D) *D. pseudoobscura*. The three characterized regulatory elements (the late element, stripes 3+7 enhancer and stripe 2 enhancer) are graphed as gray boxes in A), and the two predicted Hb sites (Hb8 to the left and Hbs1 on the right) affected by the deletions in *D. melanogaster* are indicated by black circles. Coordinates are according to a manually edited Multiz alignment of 12 *Drosophila* species.
(TIFF)

Figure S3 Effects of *Hb8Δ* alleles on viability (above) and developmental time (below). Represented are least square mean estimates for combination of eve mutation (alleles and deficiency chromosomes), balancer (Cy) or mutation carrying chromosome (DF) and sex. Developmental time was estimated as the time to eclosion.
(TIFF)

Table S1 Oligonucleotide primers used for PCR amplification, DNA sequencing and/or genotyping. Chimeric primers were used to PCR and sequence the *eve* locus, with a 5′ tag corresponding to the M13 universal sequencing primers (lowercase).
(XLS)

Table S2 Predicted Hb binding sites in the regulatory region upstream of *eve*, in 5 *Drosophila* species and the source alignments. Sheet one lists the Jaspar predicted Hb sites in *D. melanogaster* (*D.mel*), *D. simulans* (*D.sim*), *D. sechellia* (*D.sec*), *D. pseudoobscura* (*D.pse*) and *D. yakuba* (*D.yak*). Coordinates are according to a manually edited Multiz alignment of 12 *Drosophila* species. Hb8 is at 4495

and Hbs1 is at 4871. See materials and methods for details. Sheet two contains multiple alignments of the *eve* region.
(XLS)

Table S3 Conservation of binding sites in the eve stripe 3+7 enhancer. Transcription factor binding site numbering of sites follows Stanjovic et al 1989, Small et al 1996 and Yan et al 1996. Hb binding site 16 is on the opposite strand. Full species names and accession numbers are listed in material and methods. (*) indicate bases shared by two overlapping binding sites. (N/A) sites not identified in these species. Full species names and accession numbers are listed in material and methods. (*) indicate bases shared by two overlapping binding sites. (N/A) sites not identified in these species. The order reflects approximately phylogenetic relationship available on http://insects. eugenes.org/species. There is length variation in T stretch between Kni5 and Hb11c; extra 1 and 2 bases in *D. sim* and *D. gri* respectively. As these are monomorphic stretches the core binding sites are presumably not affected.
(DOC)

Table S4 Little evolutionary conservation of a putative sloppy-paired site in the *eve* stripe 3+7 enhancer. Full species names and accession numbers are listed in material and methods. Orthology of the sloppy-paired binding site region was determined by colinearity of binding sites in the stripe 3+7 region, were Hb8 and Hb9 flank the sloppy-paired binding site. Fewer than 50 bp separated Hb8 and Hb9 in all species. The exception is *D. ananassae*, were Hb8 was not detected.
(DOC)

Table S5 The presence of the *Hb8Δ* in a world wide sample of populations. A deletion specific primer, annealing to regions joined by the mutation was used in a PCR on pooled DNA (100 individuals) from each of the 51 populations. Pop: Population.
(DOC)

Table S6 Mixed model ANOVA on *eve* stripe positioning.
(DOC)

Supporting information S1 Alignment of population sequencing of a part of *evenskipped* stripes 3+7 enhancer from North Carolina, in fasta format.
(TXT)

Supporting information S2 Alignment of population sequencing of a part of the *hunchback* regulatory region from North Carolina, in fasta format.
(TXT)

Acknowledgments

Thanks to Walter Eanes, Jean Claude Walser, Peter Andolfato and Ian Dworkin for flies or DNA from flies. Thank to Casey Bergman for help with for primer design. Thanks to Einar Arnason and his staff for help with sequencing. We thank two reviewers for good comments on the manuscript.

Author Contributions

Conceived and designed the experiments: AP MK MZL. Performed the experiments: AP NW SR MZL. Analyzed the data: AP. Contributed reagents/materials/analysis tools: AP MZL. Wrote the paper: AP NW MK. Fly work: AP. Molecular work: AP NW SR. Embryo work: AP MZL.

References

1. Raff RA (1996) The Shape of Life: Genes, Development, and the Evolution of Animal Form. University Of Chicago Press. Available: http://www.amazon.com / The-Shape-Life-Development-Evolution/dp/0226702669. Accessed 21 August 2013.
2. Gould S (1977) Ontogeny and Phylogeny. Boston: Belknap Press of Harvard University Press.
3. Wray GA, Hahn MW, Abouheif E, Balhoff JP, Pizer M, et al. (2003) The evolution of transcriptional regulation in eukaryotes. Molecular biology and evolution 20: 1377–1419. Available: http://www.ncbi.nlm.nih.gov/ pubmed/ 12777501. Accessed 8 August 2013.
4. Carroll SB (2008) Evo-devo and an expanding evolutionary synthesis: a genetic theory of morphological evolution. Cell 134: 25–36. Available: http://www.ncbi.nlm.nih.gov/pubmed /18614008. Accessed 26 May 2013.
5. Payankaulam S, Li LM, Arnosti DN (2010) Transcriptional repression: conserved and evolved features. Current biology?: CB 20: R764–71. Available: http://www.pubmedcentral.nih.gov / articlerender.fcgi?artid = 3033598&tool = pmcentrez&rendertype = abstract. Accessed 10 March 2013.
6. Arnosti DN (2003) Analysis and function of transcriptional regulatory elements: insights from Drosophila. Annual review of entomology 48: 579–602. Available: http://www.ncbi.nlm.nih.gov/ pubmed/12359740. Accessed 19 August 2013.
7. Dermitzakis ET, Clark AG (2002) Evolution of transcription factor binding sites in Mammalian gene regulatory regions: conservation and turnover. Molecular biology and evolution 19: 1114–1121. Available: http://www.ncbi.nlm.nih.gov/ pubmed /12082130. Accessed 19 May 2013.
8. Ludwig MZ, Patel NH, Kreitman M (1998) Functional analysis of eve stripe 2 enhancer evolution in Drosophila: rules governing conservation and change. Development (Cambridge, England) 125: 949–958. Available: http://www.ncbi.nlm.nih.gov/pubmed /9449677. Accessed 19 March 2013.
9. Ludwig MZ, Kreitman M (1995) Evolutinary dynamics of the Enhancer region of even-skipped in Drosophila. Molecular biology and evolution 12: 1002–1011.
10. Ludwig MZ (2002) Functional evolution of noncoding DNA. Current opinion in genetics & development 12: 634–639. Available: http://www.ncbi.nlm.nih.gov/ pubmed/12433575. Accessed 19 May 2013.
11. Ludwig MZ, Palsson A, Alekseeva E, Bergman CM, Nathan J, et al. (2005) Functional evolution of a cis-regulatory module. PLoS biology 3: e93. Available: http://www.pubmedcentral.nih.gov/ articlerender.fcgi?artid = 1064851&tool = pmcentrez&rendertype = abstract. Accessed 19 March 2013.
12. Crocker J, Tamori Y, Erives A (2008) Evolution acts on enhancer organization to fine-tune gradient threshold readouts. PLoS biology 6: e263. Available: http://www.pubmedcentral.nih.gov/ articlerender.fcgi?artid = 2577699&tool = pmcentrez&rendertype = abstract. Accessed 6 August 2013.
13. Carter AJR, Wagner GP (2002) Evolution of functionally conserved enhancers can be accelerated in large populations: a population-genetic model. Proceedings Biological sciences/The Royal Society 269: 953–960. Available: http: //www.pubmedcentral.nih.gov/ articlerender.fcgi?artid = 1690979&tool = pmcentrez&rendertype = abstract. Accessed 12 March 2013.
14. Gerland U, Hwa T (2002) On the selection and evolution of regulatory DNA motifs. Journal of molecular evolution 55: 386–400. Available: http://www.ncbi.nlm.nih.gov/ pubmed/12355260. Accessed 11 March 2013.
15. Mustonen V, Lässig M (2005) Evolutionary population genetics of promoters: predicting binding sites and functional phylogenies. Proceedings of the National Academy of Sciences of the United States of America 102: 15936–15941. Available: http://www.pubmedcentral.nih.gov/ articlerender.fcgi?artid = 1276062&tool = pmcentrez&rendertype = abstract.
16. Sinha S, Siggia ED (2005) Sequence turnover and tandem repeats in cis-regulatory modules in drosophila. Molecular biology and evolution 22: 874–885. Available: http://www.ncbi.nlm.nih.gov/ pubmed/15659554. Accessed 21 August 2013.
17. Landry CR, Wittkopp PJ, Taubes CH, Ranz JM, Clark AG, et al. (2005) Compensatory cis-trans evolution and the dysregulation of gene expression in interspecific hybrids of Drosophila. Genetics 171: 1813–1822. Available: http://www.pubmedcentral.nih.gov/ articlerender.fcgi?artid = 1456106&tool = pmcentrez&rendertype = abstract. Accessed 21 August 2013.
18. Tanay A, Regev A, Shamir R (2005) Conservation and evolvability in regulatory networks: the evolution of ribosomal regulation in yeast. Proceedings of the National Academy of Sciences of the United States of America 102: 7203–7208. Available: http://www.pubmedcentral.nih.gov /articlerender.fcgi?artid = 1091753&tool = pmcentrez&rendertype = abstract. Accessed 8 May 2013.
19. Ihmels J, Bergmann S, Gerami-Nejad M, Yanai I, McClellan M, et al. (2005) Rewiring of the yeast transcriptional network through the evolution of motif usage. Science (New York, NY) 309: 938–940. Available: http://www.ncbi.nlm.nih.gov/pubmed/16081737. Accessed 8 March 2013.
20. Stone JR, Wray GA (2001) Rapid evolution of cis-regulatory sequences via local point mutations. Molecular biology and evolution 18: 1764–1770. Available: http://www.ncbi.nlm.nih.gov/pubmed/11504856. Accessed 21 August 2013.
21. Rockman M V, Hahn MW, Soranzo N, Zimprich F, Goldstein DB, et al. (2005) Ancient and recent positive selection transformed opioid cis-regulation in humans. PLoS biology 3: e387. Available: http://www.pubmedcentral.nih.gov/articlerender.fcgi?artid = 1283535&tool = pmcentrez&rendertype = abstract. Accessed 12 March 2013.
22. Kim J, He X, Sinha S (2009) Evolution of regulatory sequences in 12 Drosophila species. PLoS genetics 5: e1000330. Available: http://www.pubmedcentral.nih.

gov/articlerender.fcgi?artid = 2607023&tool = pmcentrez&rendertype = abstract. Accessed 21 August 2013.
23. Schaeffer SW (2002) Molecular population genetics of sequence length diversity in the Adh region of Drosophila pseudoobscura. Genetical research 80: 163–175. Available: http://www.ncbi.nlm.nih.gov/pubmed/12688655. Accessed 21 August 2013.
24. Comeron JM, Kreitman M (2000) The correlation between intron length and recombination in drosophila. Dynamic equilibrium between mutational and selective forces. Genetics 156: 1175–1190. Available: http://www.pubmedcentral.nih.gov/articlerender.fcgi?artid = 1461334&tool = pmcentrez&rendertype = abstract. Accessed 21 August 2013.
25. Ometto L, Stephan W, De Lorenzo D (2005) Insertion/deletion and nucleotide polymorphism data reveal constraints in Drosophila melanogaster introns and intergenic regions. Genetics 169: 1521–1527. Available: http://www.pubmedcentral.nih.gov/articlerender.fcgi?artid = 1449560&tool = pmcentrez&rendertype = abstract. Accessed 21 August 2013.
26. Leushkin E V, Bazykin G a, Kondrashov AS (2013) Strong mutational bias toward deletions in the Drosophila melanogaster genome is compensated by selection. Available: http://www.pubmedcentral.nih.gov/articlerender.fcgi?artid = 3622295&tool = pmcentrez&rendertype = abstract. Accessed 14 August 2013.
27. Goering LM, Hunt PK, Heighington C, Busick C, Pennings PS, et al. (2009) Association of orthodenticle with natural variation for early embryonic patterning in Drosophila melanogaster. Journal of experimental zoology Part B, Molecular and developmental evolution 312: 841–854. Available: http://www.pubmedcentral.nih.gov/articlerender.fcgi?artid = 2784951&tool = pmcentrez&rendertype = abstract. Accessed 21 August 2013.
28. Romano LA, Wray GA (2003) Conservation of Endo16 expression in sea urchins despite evolutionary divergence in both cis and trans-acting components of transcriptional regulation. Development (Cambridge, England) 130: 4187–4199. Available: http://www.ncbi.nlm.nih.gov/pubmed/12874137. Accessed 19 May 2013.
29. Balhoff JP, Wray GA (2005) Evolutionary analysis of the well characterized endo16 promoter reveals substantial variation within functional sites. Proceedings of the National Academy of Sciences of the United States of America 102: 8591–8596. Available: http://www.pubmedcentral.nih.gov/articlerender.fcgi?artid = 1150811&tool = pmcentrez&rendertype = abstract. Accessed 19 May 2013.
30. Garfield D, Haygood R, Nielsen WJ, Wray GA (2012) Population genetics of cis-regulatory sequences that operate during embryonic development in the sea urchin Strongylocentrotus purpuratus. Evolution & development 14: 152–167. Available: http://www.ncbi.nlm.nih.gov/pubmed/23017024. Accessed 21 August 2013.
31. Nüsslein-Volhard C, Wieschaus E (1980) Mutations affecting segment number and polarity in Drosophila. Nature 287: 795–801. Available: http://www.ncbi.nlm.nih.gov/pubmed/6776413. Accessed 17 March 2013.
32. Yan R, Small S, Desplan C, Dearolf CR, Darnell JE (1996) Identification of a Stat gene that functions in Drosophila development. Cell 84: 421–430. Available: http://www.ncbi.nlm.nih.gov/pubmed/8608596.
33. Schroeder MD, Pearce M, Fak J, Fan H, Unnerstall U, et al. (2004) Transcriptional control in the segmentation gene network of Drosophila. PLoS biology 2: E271. Available: http://www.pubmedcentral.nih.gov/articlerender.fcgi?artid = 514885&tool = pmcentrez&rendertype = abstract. Accessed 6 August 2013.
34. Jaeger J, Blagov M, Kosman D, Kozlov KN, Manu, etal. (2004) Dynamical analysis of regulatory interactions in the gap gene system of Drosophila melanogaster. Genetics 167: 1721–1737. Available: http://www.pubmedcentral.nih.gov/articlerender.fcgi?artid = 1471003&tool = pmcentrez&rendertype = abstract. Accessed 22 August 2013.
35. Jaeger J, Surkova S, Blagov M, Janssens H, Kosman D, et al. (2004) Dynamic control of positional information in the early Drosophila embryo. Nature 430: 368–371. Available: http://www.ncbi.nlm.nih.gov/pubmed/15254541. Accessed 22 August 2013.
36. Small S, Kraut R, Hoey T, Warrior R, Levine M (1991) Transcriptional regulation of a pair-rule stripe in Drosophila. Genes & development 5: 827–839. Available: http://www.ncbi.nlm.nih.gov/pubmed/2026328. Accessed 22 August 2013.
37. Small S, Arnosti DN, Levine M (1993) Spacing ensures autonomous expression of different stripe enhancers in the even-skipped promoter. Development (Cambridge, England) 119: 762–772. Available: http://www.ncbi.nlm.nih.gov/pubmed/8187640. Accessed 22 August 2013.
38. Hiromi Y, Kuroiwa A, Gehring WJ (1985) Control elements of the Drosophila segmentation gene fushi tarazu. Cell 43: 603–613. Available: http://www.ncbi.nlm.nih.gov/pubmed/3935327. Accessed 22 August 2013.
39. Ludwig MZ, Bergman C, Patel NH, Kreitman M (2000) Evidence for stabilizing selection in a eukaryotic enhancer element. Nature 403: 564–567. Available: http://www.ncbi.nlm.nih.gov/pubmed/10676967. Accessed 12 March 2013.
40. True JR, Haag ES (2001) Developmental system drift and flexibility in evolutionary trajectories. Evolution & development 3: 109–119. Available: http://www.ncbi.nlm.nih.gov/pubmed/11341673. Accessed 22 August 2013.
41. Gibson G (2000) Evolution: hox genes and the cellared wine principle. Current biology?: CB 10: R452–5. Available: http://www.ncbi.nlm.nih.gov/pubmed/10873798. Accessed 22 August 2013.
42. Palsson A, Gibson G (2004) Association between nucleotide variation in Egfr and wing shape in Drosophila melanogaster. Genetics 167: 1187–1198. Available: http://www.pubmedcentral.nih.gov/articlerender.fcgi?artid = 1470961&tool = pmcentrez&rendertype = abstract. Accessed 2 June 2013.
43. Verrelli BC, Eanes WF (2001) Clinal variation for amino acid polymorphisms at the Pgm locus in Drosophila melanogaster. Genetics 157: 1649–1663. Available: http://www.pubmedcentral.nih.gov/articlerender.fcgi?artid = 1461594&tool = pmcentrez&rendertype = abstract. Accessed 22 August 2013.
44. Walser J-C, Chen B, Feder ME (2006) Heat-shock promoters: targets for evolution by P transposable elements in Drosophila. PLoS genetics 2: e165. Available: http://www.pubmedcentral.nih.gov/articlerender.fcgi?artid = 1592238&tool = pmcentrez&rendertype = abstract. Accessed 14 August 2013.
45. Rozen S, Skaletsky H (2000) Primer3 on the WWW for general users and for biologist programmers. Methods in molecular biology (Clifton, NJ) 132: 365–386. Available: http://www.ncbi.nlm.nih.gov/pubmed/10547847. Accessed 14 August 2013.
46. Palsson A, Rouse A, Riley-Berger R, Dworkin I, Gibson G (2004) Nucleotide variation in the Egfr locus of Drosophila melanogaster. Genetics 167: 1199–1212. Available: http://www.pubmedcentral.nih.gov/articlerender.fcgi?artid = 1470963&tool = pmcentrez&rendertype = abstract. Accessed 20 August 2013.
47. Bradbury PJ, Zhang Z, Kroon DE, Casstevens TM, Ramdoss Y, et al. (2007) TASSEL: software for association mapping of complex traits in diverse samples. Bioinformatics (Oxford, England) 23: 2633–2635. Available: http://www.ncbi.nlm.nih.gov/pubmed/17586829. Accessed 23 May 2013.
48. R Development Core Team (2011) R: A language and environment for statistical computing. R Foundation for Statistical Computing. Available: http://www.r-project.org/.
49. Rozas J, Sánchez-DelBarrio JC, Messeguer X, Rozas R (2003) DnaSP, DNA polymorphism analyses by the coalescent and other methods. Bioinformatics (Oxford, England) 19: 2496–2497. Available: http://www.ncbi.nlm.nih.gov/pubmed/14668244. Accessed 22 August 2013.
50. Hudson RR, Bailey K, Skarecky D, Kwiatowski J, Ayala FJ (1994) EVIDENCE FOR POSITIVE SELECTION IN THE SUPEROXIDE-DISMUTASE (SOD) REGION OF DROSOPHILA-MELANOGASTER. Genetics 136: 1329–1340. Available: http://www.pubmedcentral.nih.gov/articlerender.fcgi?artid = 1205914&tool = pmcentrez&rendertype = abstract. Accessed 21 May 2013.
51. Bray N, Dubchak I, Pachter L (2003) AVID: A global alignment program. Genome research 13: 97–102. Available: http://www.pubmedcentral.nih.gov/articlerender.fcgi?artid = 430967&tool = pmcentrez&rendertype = abstract. Accessed 15 August 2013.
52. Nicholas KB, Nicholas HB Jr, Deerfield DWI (1997) GeneDoc: Analysis and Visualization of Genetic Variation. EMBNEW NEWS. Available: Http://www.psc.edu/biomed/genedoc.
53. Halfon MS, Gallo SM, Bergman CM (2008) REDfly 2.0: an integrated database of cis-regulatory modules and transcription factor binding sites in Drosophila. Nucleic acids research 36: D594–8. Available: http://www.pubmedcentral.nih.gov/articlerender.fcgi?artid = 2238825&tool = pmcentrez&rendertype = abstract. Accessed 22 August 2013.
54. Griffith OL, Montgomery SB, Bernier B, Chu B, Kasaian K, et al. (2008) ORegAnno: an open-access community-driven resource for regulatory annotation. Nucleic acids research 36: D107–13. Available: http://www.pubmedcentral.nih.gov/articlerender.fcgi?artid = 2239002&tool = pmcentrez&rendertype = abstract. Accessed 18 August 2013.
55. Stanojević D, Hoey T, Levine M (1989) Sequence-specific DNA-binding activities of the gap proteins encoded by hunchback and Krüppel in Drosophila. Nature 341: 331–335. Available: http://www.ncbi.nlm.nih.gov/pubmed/2507923. Accessed 22 August 2013.
56. Sandelin A, Alkema W, Engström P, Wasserman WW, Lenhard B (2004) JASPAR: an open-access database for eukaryotic transcription factor binding profiles. Nucleic acids research 32: D91–4. Available: http://www.pubmedcentral.nih.gov/articlerender.fcgi?artid = 308747&tool = pmcentrez&rendertype = abstract. Accessed 21 January 2014.
57. Rasband WS (1997) Image J. Available: http://imagej.nih.gov/ij/.
58. Institute S (2001) SAS.
59. Small S, Blair a, Levine M (1996) Regulation of two pair-rule stripes by a single enhancer in the Drosophila embryo. Developmental biology 175: 314–324. Available: http://www.ncbi.nlm.nih.gov/pubmed/8626035.
60. Robin C, Lyman RF, Long AD, Langley CH, Mackay TFC (2002) hairy: A quantitative trait locus for drosophila sensory bristle number. Genetics 162: 155–164. Available: http://www.pubmedcentral.nih.gov/articlerender.fcgi?artid = 1462234&tool = pmcentrez&rendertype = abstract. Accessed 22 August 2013.
61. Clark AG, Eisen MB, Smith DR, Bergman CM, Oliver B, et al. (2007) Evolution of genes and genomes on the Drosophila phylogeny. Nature 450: 203–218. Available: http://www.ncbi.nlm.nih.gov/pubmed/17994087. Accessed 8 August 2013.
62. Andrioli LPM, Vasisht V, Theodosopoulou E, Oberstein A, Small S (2002) Anterior repression of a Drosophila stripe enhancer requires three position-specific mechanisms. Development (Cambridge, England) 129: 4931–4940. Available: http://www.ncbi.nlm.nih.gov/pubmed/12397102. Accessed 22 August 2013.
63. Fujioka M, Yusibova GL, Patel NH, Brown SJ, Jaynes JB (2002) The repressor activity of Even-skipped is highly conserved, and is sufficient to activate engrailed and

to regulate both the spacing and stability of parasegment boundaries. Development (Cambridge, England) 129: 4411–4421. Available: http://www.pubmedcentral.nih.gov/articlerender.fcgi?artid = 2709299&tool = pmcentrez&rendertype = abstract. Accessed 22 August 2013.

64. Nüsslein-Volhard C, Kluding H, Jürgens G (1985) Genes affecting the segmental subdivision of the Drosophila embryo. Cold Spring Harbor symposia on quantitative biology 50: 145–154. Available: http://www.ncbi.nlm.nih.gov/pubmed/3868475. Accessed 22 August 2013.
65. Moses AM, Pollard DA, Nix DA, Iyer VN, Li X-Y, et al. (2006) Large-scale turnover of functional transcription factor binding sites in Drosophila. PLoS computational biology 2: e130. Available: http://www.pubmedcentral.nih.gov/articlerender.fcgi?artid = 1599766&tool = pmcentrez&rendertype = abstract. Accessed 4 March 2013.
66. He BZ, Holloway AK, Maerkl SJ, Kreitman M (2011) Does positive selection drive transcription factor binding site turnover? A test with Drosophila cis-regulatory modules. PLoS genetics 7: e1002053. Available: http://www.pubmedcentral.nih.gov/articlerender.fcgi?artid = 3084208&tool = pmcentrez&rendertype = abstract. Accessed 21 May 2013.
67. Stranger BE, Forrest MS, Dunning M, Ingle CE, Beazley C, et al. (2007) Relative impact of nucleotide and copy number variation on gene expression phenotypes. Science (New York, NY) 315: 848–853. Available: http://www.pubmedcentral.nih.gov/articlerender.fcgi?artid = 2665772&tool = pmcentrez&rendertype = abstract. Accessed 27 February 2013.
68. Chan YF, Marks ME, Jones FC, Villarreal G, Shapiro MD, et al. (2010) Adaptive evolution of pelvic reduction in sticklebacks by recurrent deletion of a Pitx1 enhancer. Science (New York, NY) 327: 302–305. Available: http://apps.webofknowledge.com/full_record.do?product = WOS&search_mode = GeneralSearch&qid = 10&SID = Y196NGp9aKnAOdl3mGB&page = 1&doc = 1. Accessed 30 May 2013.
69. Kirst M, Myburg AA, De León JPG, Kirst ME, Scott J, et al. (2004) Coordinated genetic regulation of growth and lignin revealed by quantitative trait locus analysis of cDNA microarray data in an interspecific backcross of eucalyptus. Plant physiology 135: 2368–2378. Available: http://www.pubmedcentral.nih.gov/articlerender.fcgi?artid = 520804&tool = pmcentrez&rendertype = abstract. Accessed 22 August 2013.
70. Brem RB, Kruglyak L (2005) The landscape of genetic complexity across 5,700 gene expression traits in yeast. Proceedings of the National Academy of Sciences of the United States of America 102: 1572–1577. Available: http://www.pubmedcentral.nih.gov/articlerender.fcgi?artid = 547855&tool = pmcentrez&rendertype = abstract. Accessed 22 August 2013.
71. Emilsson V, Thorleifsson G, Zhang B, Leonardson AS, Zink F, et al. (2008) Genetics of gene expression and its effect on disease. Nature 452: 423–428. Available: http://www.ncbi.nlm.nih.gov/pubmed/18344981. Accessed 27 February 2013.
72. Rockman M V, Wray GA (2002) Abundant raw material for cis-regulatory evolution in humans. Molecular biology and evolution 19: 1991–2004. Available: http://www.ncbi.nlm.nih.gov/pubmed/12411608. Accessed 30 April 2013.
73. Kim A-R, Martinez C, Ionides J, Ramos AF, Ludwig MZ, et al. (2013) Rearrangements of 2.5 kilobases of noncoding DNA from the Drosophila even-skipped locus define predictive rules of genomic cis-regulatory logic. PLoS genetics 9: e1003243. Available: http://dx.plos.org/10.1371/journal.pgen.1003243. Accessed 16 September 2013.
74. Arnosti DN, Barolo S, Levine M, Small S (1996) The eve stripe 2 enhancer employs multiple modes of transcriptional synergy. Development (Cambridge, England) 122: 205–214. Available: http://www.ncbi.nlm.nih.gov/pubmed/8565831.
75. Shimell MJ, Peterson AJ, Burr J, Simon JA, O'Connor MB (2000) Functional analysis of repressor binding sites in the iab-2 regulatory region of the abdominal-A homeotic gene. Developmental biology 218: 38–52. Available: http://www.ncbi.nlm.nih.gov/pubmed/10644409. Accessed 23 August 2013.
76. Kalinka AT, Varga KM, Gerrard DT, Preibisch S, Corcoran DL, et al. (2010) Gene expression divergence recapitulates the developmental hourglass model. Nature 468: 811–814. Available: http://www.ncbi.nlm.nih.gov/pubmed/21150996. Accessed 27 May 2013.
77. Lott SE, Villalta JE, Schroth GP, Luo S, Tonkin LA, et al. (2011) Noncanonical compensation of zygotic X transcription in early Drosophila melanogaster development revealed through single-embryo RNA-seq. PLoS biology 9: e1000590. Available: http://www.pubmedcentral.nih.gov/articlerender.fcgi?artid = 3035605&tool = pmcentrez&rendertype = abstract. Accessed 6 March 2013.
78. Lott SE, Kreitman M, Palsson A, Alekseeva E, Ludwig MZ (2007) Canalization of segmentation and its evolution in Drosophila. Proceedings of the National Academy of Sciences of the United States of America 104: 10926–10931. Available: http://www.pubmedcentral.nih.gov/articlerender.fcgi?artid = 1891814&tool = pmcentrez&rendertype = abstract. Accessed 13 August 2013.
79. Houchmandzadeh B, Wieschaus E, Leibler S (2002) Establishment of developmental precision and proportions in the early Drosophila embryo. Nature 415: 798–802. Available: http://www.ncbi.nlm.nih.gov/pubmed/11845210. Accessed 23 August 2013.
80. Gregor T, Bialek W, de Ruyter van Steveninck RR, Tank DW, Wieschaus EF (2005) Diffusion and scaling during early embryonic pattern formation. Proceedings of the National Academy of Sciences of the United States of America 102: 18403–18407. Available: http://www.pubmedcentral.nih.gov/articlerender.fcgi?artid = 1311912&tool = pmcentrez&rendertype = abstract. Accessed 23 August 2013.
81. Surkova S, Kosman D, Kozlov K, Manu, Myasnikova E, et al. (2008) Characterization of the Drosophila segment determination morphome. Developmental Biology 313: 844–862. Available: http://www.sciencedirect.com/science/article/pii/S0012160607014662. Accessed 16 September 2013.
82. Cleary MD, Doe CQ (2006) Regulation of neuroblast competence: multiple temporal identity factors specify distinct neuronal fates within a single early competence window. Genes & development 20: 429–434. Available: http://www.pubmedcentral.nih.gov/articlerender.fcgi?artid = 1369045&tool = pmcentrez&rendertype = abstract. Accessed 10 August 2013.
83. Doe CQ, Smouse D, Goodman CS (1988) Control of neuronal fate by the Drosophila segmentation gene even-skipped. Nature 333: 376–378. Available: http://www.ncbi.nlm.nih.gov/pubmed/3374572. Accessed 28 August 2013.
84. Wagner A (2008) Neutralism and selectionism: a network-based reconciliation. Nature reviews Genetics 9: 965–974. Available: http://www.ncbi.nlm.nih.gov/pubmed/18957969. Accessed 16 August 2013.
85. Hülskamp M, Pfeifle C, Tautz D (1990) A morphogenetic gradient of hunchback protein organizes the expression of the gap genes Krüppel and knirps in the early Drosophila embryo. Nature 346: 577–580. Available: http://www.ncbi.nlm.nih.gov/pubmed/2377231. Accessed 13 August 2013.
86. Zuo P, Stanojević D, Colgan J, Han K, Levine M, et al. (1991) Activation and repression of transcription by the gap proteins hunchback and Krüppel in cultured Drosophila cells. Genes & development 5: 254–264. Available: http://www.ncbi.nlm.nih.gov/pubmed/1671661. Accessed 29 August 2013.
87. Papatsenko D, Levine MS (2008) Dual regulation by the Hunchback gradient in the Drosophila embryo. Proceedings of the National Academy of Sciences of the United States of America 105: 2901–2906. Available: http://www.pubmedcentral.nih.gov/articlerender.fcgi?artid = 2268557&tool = pmcentrez&rendertype = abstract.
88. Arnosti DN (2011) Transcriptional repressors: shutting off gene expression at the source affects developmental dynamics. Current biology?: CB 21: R859–60. Available: http://www.ncbi.nlm.nih.gov/pubmed/22032193. Accessed 27 April 2013.
89. Stern DL, Orgogozo V (2008) The loci of evolution: how predictable is genetic evolution? Evolution; international journal of organic evolution 62: 2155–2177. Available: http://www.pubmedcentral.nih.gov/articlerender.fcgi?artid = 2613234&tool = pmcentrez&rendertype = abstract. Accessed 9 August 2013.
90. Jaeger J, Reinitz J (2006) On the dynamic nature of positional information. BioEssays 28: 1102–1111. Available: http://www.ncbi.nlm.nih.gov/pubmed/17041900. Accessed 9 April 2013.
91. Wunderlich Z, DePace AH (2011) Modeling transcriptional networks in Drosophila development at multiple scales. Current opinion in genetics & development 21: 711–718. Available: http://www.ncbi.nlm.nih.gov/pubmed/21889888. Accessed 18 August 2013.
92. Segal E, Raveh-Sadka T, Schroeder M, Unnerstall U, Gaul U (2008) Predicting expression patterns from regulatory sequence in Drosophila segmentation. Nature 451: 535–540. Available: http://www.ncbi.nlm.nih.gov/pubmed/18172436. Accessed 6 August 2013.
93. Manu, Ludwig MZ, Kreitman M (2013) Sex-specific pattern formation during early Drosophila development. Genetics 194: 163–173. Available: http://www.ncbi.nlm.nih.gov/pubmed/23410834.
94. Ni X, Zhang YE, Nègre N, Chen S, Long M, et al. (2012) Adaptive evolution and the birth of CTCF binding sites in the Drosophila genome. PLoS biology 10: e1001420. Available: http://www.pubmedcentral.nih.gov/articlerender.fcgi?artid = 3491045&tool = pmcentrez&rendertype = abstract. Accessed 13 August 2013.
95. Barrière A, Gordon KL, Ruvinsky I (2012) Coevolution within and between regulatory loci can preserve promoter function despite evolutionary rate acceleration. PLoS genetics 8: e1002961. Available: http://www.pubmedcentral.nih.gov/articlerender.fcgi?artid = 3447958&tool = pmcentrez&rendertype = abstract. Accessed 21 August 2013.
